# A distinct form of fat fibrosis is linked to insulin resistance in people with HIV

**DOI:** 10.1172/jci.insight.195922

**Published:** 2026-06-30

**Authors:** Diana L. Alba, Alaa Abdellatif, Moon Kyung Choi, Stephen M. Brown Mayfield, Thuy An T. Pham, David I. Berrios, Antonio E. Rodriguez, Marin Ewing, Tony R. Figueroa, Judy Gonzalez-Vargas, Ningyan Zhang, Zhiqiang An, Dawei Bu, Steven G. Deeks, Philipp E. Scherer, Peter W. Hunt, Suneil K. Koliwad

**Affiliations:** 1 Division of Endocrinology, Diabetes and Metabolism, Department of Medicine, Zuckerberg San Francisco General Hospital and UCSF, San Francisco, California, USA.; 2 UCSF Center for AIDS Research (CFAR), San Francisco, California, USA.; 3 UCSF AIDS Research Institute, San Francisco, California, USA.; 4 UCSF Data Science CoLab, San Francisco, California, USA.; 5 Division of Endocrinology & Metabolism, UCSF, San Francisco, California, USA.; 6 UCSF Diabetes Center, San Francisco, California, USA.; 7 Division of HIV, Infectious Diseases, and Global Medicine, UCSF, San Francisco, California, USA.; 8 Texas Therapeutics Institute, Brown Foundation Institute of Molecular Medicine, University of Texas Health Science Center at Houston, Houston, Texas, USA.; 9 Touchstone Diabetes Center, University of Texas Southwestern Medical Center, Dallas, Texas, USA.; 10 Division of Experimental Medicine, Department of Medicine, Zuckerberg San Francisco General Hospital and UCSF, San Francisco, California, USA.

**Keywords:** AIDS/HIV, Endocrinology, Adipose tissue, Fibrosis, Insulin

## Abstract

**BACKGROUND:**

Despite antiretroviral therapy (ART), people with HIV (PWH) are at heightened risk for insulin resistance (IR) and type 2 diabetes (T2D). Subcutaneous adipose tissue (SAT) fibrosis contributes to metabolic disease, but its role in IR among PWH is unknown. We investigated the relationship between SAT fibrosis and IR in PWH, along with transcriptional signatures to distinguish it from SAT fibrosis due to obesity.

**METHODS:**

We analyzed body composition and SAT fibrosis (hydroxyproline) in 46 PWH and 74 people without HIV (PWoH), excluding individuals with T2D. We examined fibrosis-related gene transcription in the SAT using a targeted panel and measured plasma endotrophin, a marker of extracellular matrix (ECM) remodeling.

**RESULTS:**

PWH had substantially more SAT fibrosis than PWoH, notably in nonobese individuals. Moreover, SAT fibrosis in these PWH was strongly associated with IR, independent of prior legacy ART or ongoing integrase strand inhibitor treatment. This SAT fibrosis was highlighted by a distinct transcriptional pattern marked by upregulation of *COL14A1*, key immune-related genes (e.g., *CCL4*, *NLRP3*), and pathways governing ECM remodeling and immune activation, as well as downregulation of thermogenic, lipid metabolic, and insulin signaling pathways. Plasma endotrophin levels were also elevated in PWH and correlated independently with SAT fibrosis.

**CONCLUSION:**

SAT fibrosis was associated with IR independent of obesity in PWH and was mirrored by circulating endotrophin levels, offering a plausible noninvasive biomarker for early intervention. The distinct transcriptional signature of HIV-associated SAT fibrosis highlights candidate mechanisms that may underlie metabolic risk and offer therapeutic avenues in this population.

**TRIAL REGISTRATION:**

ClinicalTrials.gov NCT03022682.

**FUNDING:**

R01DK141041; R01DK112304; R56DK133997; K08DK124679; T32DK007418; P30DK098722; P30AI027763; Robert Wood Johnson Foundation; Harold Amos Medical Faculty Development Program.

## Abstract 2

People with HIV develop a distinct type of fat tissue fibrosis linked to insulin resistance, even when lean, pointing to new ways to assess metabolic risk.

## Introduction

People living with human immunodeficiency virus (HIV) infection (PWH) have higher rates of metabolic diseases, including cardiovascular disease, hypertension, and type 2 diabetes mellitus (T2D) compared with people without HIV (PWoH) (1, 2). This increased risk is not confined to untreated HIV. Indeed, several studies show that even people whose HIV infection is durably controlled by antiretroviral therapy (ART) face a 2- to 4-fold higher risk of incident T2D than PWoH, emphasizing that metabolic complications are a concern for PWH, despite effective viral suppression (1, 3–5).

Beyond the well-established drivers of insulin resistance (IR) and T2D in the general population, unique factors amplify disease risk for PWH. One is the use of particular ART regimens. For example, certain protease inhibitors, such as indinavir, lopinavir, and ritonavir reversibly induce IR by inhibiting glucose transport via GLUT4 (6–10). Legacy nucleoside reverse transcriptase inhibitors (NRTIs), including “d-drugs” such as stavudine (D4T) and zidovudine (AZT), disrupt glucose metabolism and promote the loss of adipose tissue (AT) mass within the subcutaneous compartment (SAT) along with a compensatory increase in visceral AT (VAT) mass. This fat redistribution, which independently increases T2D risk, is termed HIV-associated lipodystrophy (11, 12). Components of modern ART regimens continue to be examined for potential metabolic effects. Integrase strand transfer inhibitors (INSTIs), for example, were shown to blunt the capacity of adipocyte progenitors to differentiate into metabolically healthy, thermogenic “beige” adipocytes (13) and have been associated with SAT fibrosis in both PWH and nonhuman primate models of HIV (14). While INSTI-based ART is also linked to a higher T2D risk than comparator regimens in large cohorts, there remains disagreement as to whether this is independent of differences in BMI (15, 16).

Importantly, however, the prevalence of T2D among PWH has risen progressively despite substantial shifts in ART composition over the past few decades, underscoring the potential role of HIV itself in driving metabolic dysfunction. Specifically, there is growing recognition that HIV infection directly affects the metabolic function and health of AT. Recent studies highlight ATs as potential reservoirs for HIV, and shifts in AT immune cell composition have been documented in PWH (17, 18). These findings have prompted the emerging concept that AT dysfunction in PWH encompasses both ART effects and also complex HIV-specific influences on AT physiology and inflammation that may be distinct from what PWoH experience in response to worsening obesity.

Notably, AT dysfunction is an established driver of IR and T2D in PWoH (19–21). Obesity induces AT remodeling, including adipocyte hypertrophy, immune cell infiltration, fibrosis, and hypoxia, each of which is associated with the development of IR. For example, hypertrophic adipocytes excessively release free fatty acids and proinflammatory cytokines such as tumor necrosis factor-α (TNF-α) and IL-6, which are implicated in disrupting insulin signaling and contributing to systemic inflammation (20, 22). Obesity-associated extracellular matrix (ECM) accumulation results in AT fibrosis, a pathological process that is associated with inflammation and postulated to drive AT dysfunction by reducing its structural plasticity and metabolic flexibility (23). For example, SAT fibrosis in PWoH is correlated with the expansion of VAT mass, which, in turn, correlates positively with IR and inversely with the capacity to lose weight following bariatric surgery (24–28). At the cellular level, organellar dysfunction in adipocytes further amplifies these disruptions, highlighting the concept that obesity collectively impacts AT metabolic health across multiple anatomical scales (29–31). Whereas systemic inflammation was shown to persist in PWH despite effective viral suppression (32), the role of SAT fibrosis in the metabolic health of PWH remains poorly understood. Focusing on this gap in knowledge may uncover potential therapeutic targets to mitigate the alarming rise in T2D rates among PWH.

Here, we leverage a powerful multiethnic cohort of both PWH and uninfected individuals spanning a wide range of adiposity, allowing us to comprehensively analyze SAT fibrosis in PWH and examine its relationship with IR. Using transcriptomic analyses, we identify molecular pathways in the SAT that are linked to IR in PWH even in the absence of obesity or T2D, an approach that unveils potential early markers of metabolic dysfunction. Our analyses also isolate the effects of HIV away from ART composition. In all, we show that SAT fibrosis is independently associated with IR in PWH, with implications for targeted monitoring and interventions to reduce T2D burden in this high-risk group.

## Results

### Participant characteristics.

We studied 120 participants, including 74 PWoH and 46 PWH, summarized in Table 1. All the PWH maintained plasma HIV RNA levels below the detectable threshold for a minimum of 1 year before enrollment. The 2 groups were well matched for key clinical parameters related to glucose homeostasis, including fasting glucose and insulin levels, Homeostatic Model Assessment for Insulin Resistance (HOMA-IR), and hemoglobin A1c (HbA1c), reflecting our sampling strategy. Similarly, the 2 groups had a comparable age, mean BMI, and total body fat percentage (%BF), as measured using dual-energy x-ray absorptiometry (DXA). The PWH group included a higher proportion of males and Black participants compared with the PWoH group (both *P* < 0.01), consistent with the broader demographic features of PWH in the San Francisco Bay Area, where Black individuals are disproportionately affected by HIV (33). Approximately one-third of PWH (32%) had prior exposure to legacy d-drugs such as stavudine (d4T), didanosine (ddI), or zalcitabine (ddC), or related early-generation NRTIs such as zidovudine (AZT), either alone or in combination regimens such as Combivir or Trizivir.

In unadjusted analyses, PWH demonstrated a modest,nonsignificant trend toward greater visceral adiposity (%VAT/total body fat). However, multivariable linear regression did not support an independent relationship between HIV status and visceral adiposity (*P* = 0.37), although increasing age did predict VAT accrual (*P* < 0.001), consistent with established knowledge for humans in general (Supplemental Table 1; supplemental material available online with this article; https://doi.org/10.1172/jci.insight.195922DS1). Together, these results reassured us that our subsequent analyses were unlikely to be confounded by significant HIV-dependent differences in visceral adiposity between groups.

### PWH have excess SAT fibrosis, independent of overall adiposity.

**Figure 1:**
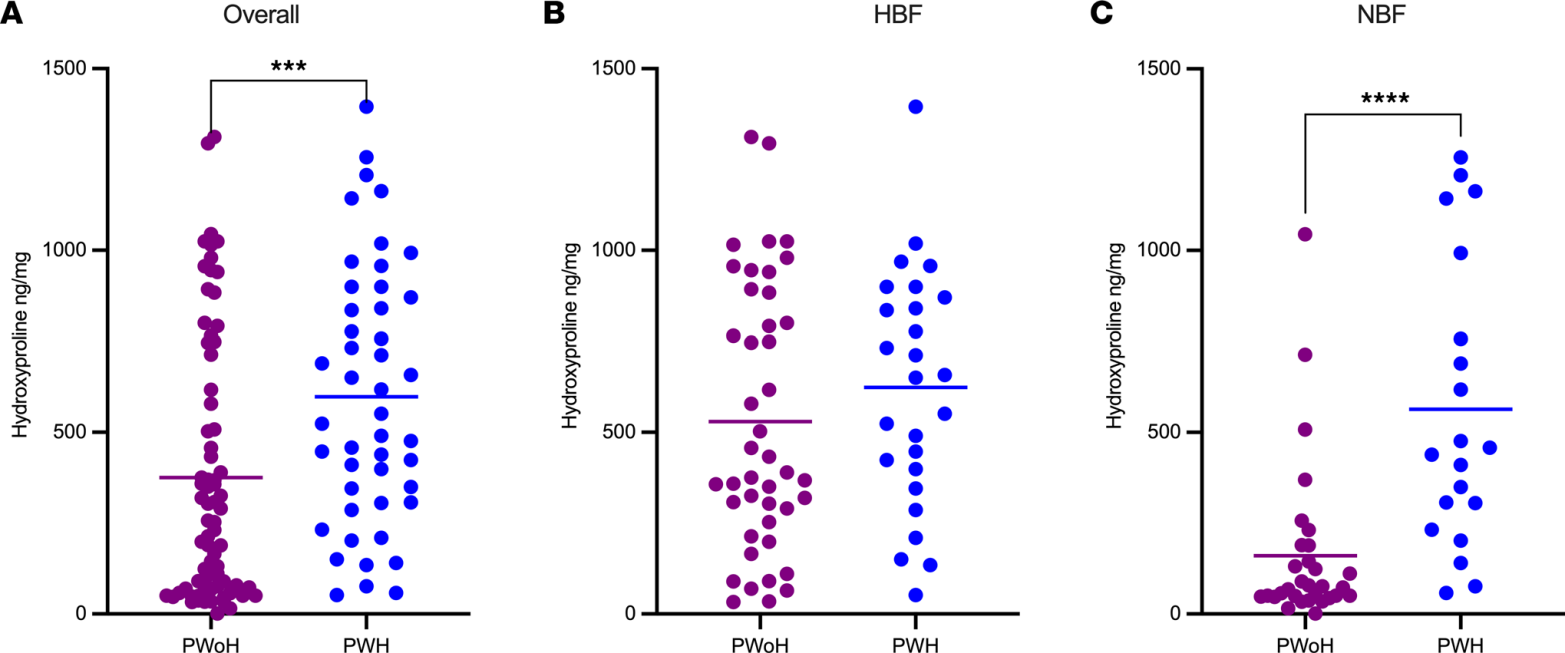
PWH have increased SAT fibrosis versus PWoH, particularly in those with normal %BF (NBF). Hydroxyproline (HYP) content (ng/mg) in SAT was compared between people without HIV (PWoH; purple) and people with HIV (PWH; blue). (**A**) In the overall cohort (PWoH, *n* = 74; PWH, *n* = 46), SAT HYP content was significantly higher in PWH than PWoH (*P* = 0.0004). (**B**) Subgroup analysis for participants with a high %BF (HBF; PWoH, *n* = 43; PWH, *n* = 26) showing no difference in SAT HYP between groups (*P* = 0.34). (**C**) Among participants with NBF, SAT HYP content was significantly elevated in PWH compared with PWoH (PWoH, *n* = 31; PWH, *n* = 20), (*P* < 0.0001). Differences between the 2 groups were analyzed by Mann-Whitney *U* test. *** *P* < 0.001, **** *P* < 0.0001.

We and others have shown that SAT fibrosis is closely linked to the development of IR, a major driver of T2D, in humans (24, 34, 35). Given the elevated metabolic risk of PWH, we assessed SAT fibrosis by quantifying levels of hydroxyproline (HYP), a key component of collagen and a validated marker of ECM remodeling (36), in SAT samples from our study participants. In unadjusted analyses, PWH had substantially higher SAT HYP levels than PWoH (Figure 1A). To determine whether this association reflects HIV status rather than differences in demographic characteristics or body composition, we performed multivariable linear regression on the data from both PWH and PWoH, adjusting for sex, age, race, and body fat percentage (%BF). Even after adjusting for these variables, HIV status remained independently associated with higher SAT HYP levels (*P* = 0.002). Male sex and %BF were also independently associated with higher SAT HYP levels, whereas age and race were not (Supplemental Table 2 and Supplemental Figure 1A). Together, these data support HIV status as an independent determinant of SAT fibrosis.

To assess the extent to which the increased SAT fibrosis we saw among PWH was a function of either ART exposure or adiposity, we performed a separate multivariable linear regression analysis that was restricted to PWH. This model evaluated associations between SAT HYP levels and legacy d-drug exposure, current INSTI use, CD4^+^ T cell count at enrollment, and %BF (Supplemental Table 3). INSTI use was included because INSTI-based regimens, common in modern ART, are associated with weight gain and could potentially promote SAT fibrosis in PWH (37). Notably, we found that neither d-drug exposure, INSTI use, CD4 count, nor %BF drove the increased SAT HYP levels we saw in PWH, indicating that the development of SAT fibrosis in PWH is not simply due to legacy ART exposure, ongoing INSTI use, or increasing adiposity. Moreover, the inclusion of sex, age, and self-identified race in this PWH-restricted model did not substantively alter effect estimates or statistical inference for the primary covariates in sensitivity analyses (Supplemental Table 4). Given the sample size and the lack of any independent associations found in these multivariable analyses, the final model did not retain these added variables in order to avoid overfitting. Together, these findings indicate that HIV infection is independently associated with increased SAT fibrosis, while the variability in SAT fibrosis among PWH is not driven by either key ART classes implicated in the literature as potential confounders, notable demographic factors, or degree of adiposity.

Given that increasing adiposity and obesity are both associated with the development of SAT fibrosis in the general population (24–26), we next performed a subgroup analysis after classifying the study participants into groups based on %BF. We chose %BF instead of BMI, as BMI does not reliably distinguish between fat mass and lean mass and may therefore misclassify adiposity, particularly across sex and racial groups (38). In contrast, %BF provides a more direct measure of total adiposity and has been shown to more closely reflect metabolic disease risk than BMI (38–40). We used %BF values of greater than or equal to 25% in male participants and greater than or equal to 35% in female participants to define obesity, aligning with established cutoffs in the field (41–43).

Accordingly, men were classified as having either “normal adiposity” (NBF, %BF less than 25%) or “high adiposity” (HBF, %BF greater than or equal to 25%), while females were classified as NBF if their %BF was less than 35% or as HBF if %BF was greater than or equal to 35%. Clinical and demographic characteristics for these 4 subgroups are summarized in Supplemental Table 5. In unadjusted analyses, SAT HYP levels were similar between PWH and PWoH with HBF (Figure 1B). By contrast, SAT HYP levels were markedly higher in PWH than in PWoH when the comparison was confined to individuals with NBF (*P* < 0.0001; Figure 1C).

Multivariable linear regression analyses adjusting for sex, age, race, and %BF validated the unadjusted findings, with HIV status remaining independently associated with higher SAT HYP levels among individuals with NBF, but not those with HBF (Supplemental Figure 1, B and C; regression coefficients and adjusted values are provided in the Supporting Data Values file). Together, these findings indicate that elevated SAT fibrosis in PWH is not driven by excess adiposity and support instead the presence of excess, obesity-independent SAT fibrosis in this population that is prominent even in relatively lean individuals.

### Differential relationships between SAT fibrosis, adiposity, and insulin resistance in PWH.

**Figure 2:**
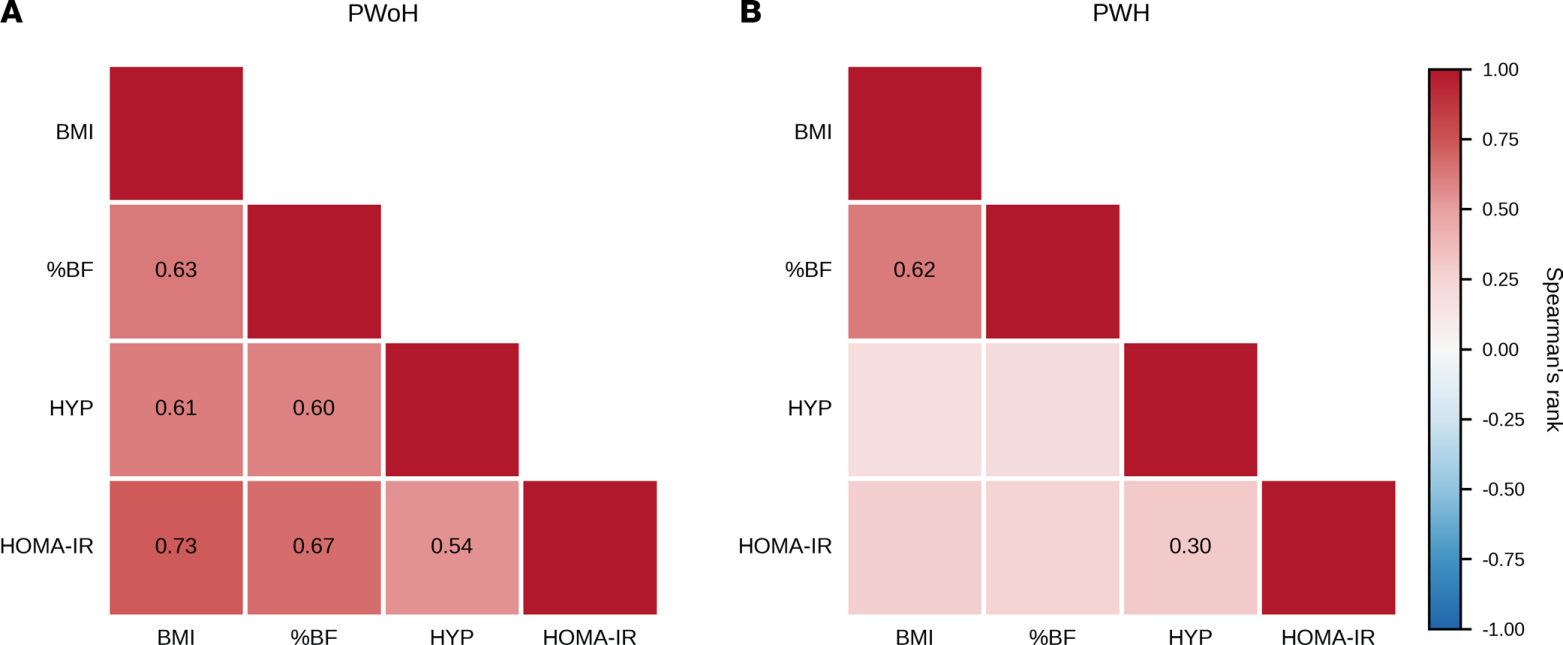
Adjusted associations between adiposity, SAT fibrosis, and IR in PWoH and PWH. Heatmaps depict partial Spearman rank-order correlations among BMI, total %BF, SAT HYP content, and IR as assessed by HOMA-IR. Panel A shows correlations in PWoH; (*n* = 73), and Panel B shows the same correlations in PWH (*n* = 46). Correlations were calculated separately within each group and adjusted for age, sex, race, and exposure to legacy d-drugs. Only statistically significant partial Spearman correlation coefficients (ρ; *P* < 0.05) are displayed. HYP: Hydroxyproline

To examine whether the increased SAT fibrosis in PWH might promote glycemic dysregulation, we evaluated relationships between SAT HYP content, adiposity, and IR using partial Spearman correlation analyses adjusted for age, sex, race, and prior exposure to legacy forms of ART. IR was assessed using HOMA-IR, a widely used clinical index of impaired insulin action (44, 45). Consistent with prior work from our group (24) and others (46, 47), increasing SAT fibrosis in PWoH correlated positively with increasing adiposity, as assessed by either BMI or %BF, and concomitantly with worsening IR (Figure 2A). In PWH, by contrast, the level of SAT fibrosis did not correlate with either BMI or %BF (Figure 2B), indicating that fibrotic SAT remodeling in this population is independent of overall adiposity. Moreover, neither BMI nor %BF were significantly associated with HOMA-IR in PWH. Remarkably, however, SAT fibrosis in PWH correlated positively with IR despite the lack of association between markers of adiposity and IR in this group, strongly suggesting that the positive correlation between SAT fibrosis and IR in PWH is independent of adiposity.

To further delineate how HIV status and adiposity modify the relationship between SAT fibrosis and IR, participants were again stratified into 4 groups (PWoH-NBF, PWoH-HBF, PWH-NBF, and PWH-HBF) using the same sex-specific %BF thresholds described above. Partial Spearman correlation analyses were then performed within each group, again adjusting for age, sex, race, and prior exposure to legacy d-drugs (Supplemental Figure 2). Among PWoH, SAT HYP levels correlated positively with both HOMA-IR and BMI in both the HBF group (ρ = 0.46 and ρ = 0.65, respectively; *P* < 0.01) and the NBF group (ρ = 0.44 and ρ = 0.65, respectively; *P* < 0.01). By contrast, SAT HYP levels in PWH were not correlated with BMI or %BF in either the NBF or HBF group. Notably, however, SAT HYP levels in PWH were strongly associated with HOMA-IR among PWH in the NBF group (ρ = 0.61, *P* < 0.05), whereas no further association between HIV and SAT HYP levels were noted in PWH who were also obese (PWH-HBF). Together, these findings indicate that, whereas SAT fibrosis in PWoH tracks both with increasing adiposity and with worsening IR across adiposity strata, consistent with established obesity-related mechanisms, SAT fibrosis in PWH is uncoupled from overall adiposity and is associated with IR even in individuals with a relatively low %BF, supporting an adiposity-independent relationship between SAT fibrosis and metabolic dysfunction in this population.

### PWH have a distinct fibrosis-associated gene transcriptional pattern in the SAT.

Given that SAT fibrosis correlated strongly with IR in PWH even in the absence of obesity or any correlation with increasing adiposity, we hypothesized that SAT fibrosis in PWH may be driven by molecular pathways that are distinct from those engaged in the setting of obesity-associated SAT fibrosis in PWoH. To test this, we profiled SAT samples using a NanoString targeted transcriptomic panel of 772 genes selected a priori for known roles in tissue fibrosis (Bruker Spatial Biology), allowing sensitive detection of coordinated, pathway-level transcriptional differences. Because this analysis was hypothesis-driven and restricted to a predefined gene set already associated with tissue fibrosis (48, 49), differential expression was assessed using stringent nominal *P*-value thresholds (*P* < 0.01) rather than genome-wide false discovery rate (FDR) correction, which is designed for unbiased, high-dimensional discovery screens. Applying FDR adjustment to a targeted panel of curated genes could be overly conservative, as the a priori biological constraint substantially reduces the effective multiple testing burden compared with unbiased transcriptomic screens. This approach is consistent with the manufacturer’s analytical recommendations and multiple prior studies employing targeted fibrosis panels (48, 50–54).

**Figure 3:**
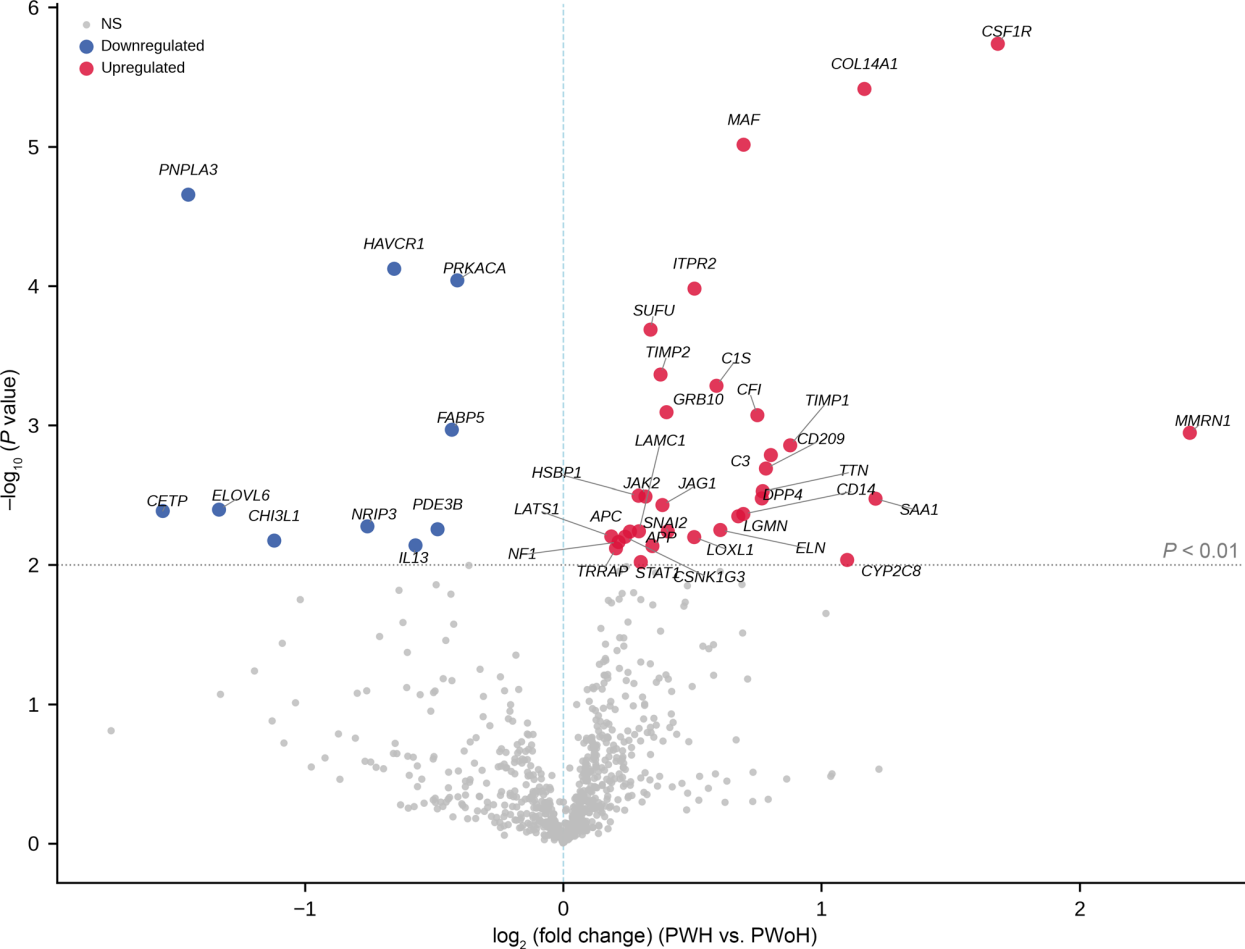
PWH have a distinct transcriptional pattern of fibrosis-associated gene expression in the SAT. Volcano plot of differential gene expression (DGE) in the SAT of PWH (*n* = 37) versus PWoH (*n* = 38) using the NanoString Human Fibrosis panel. The *x* axis shows log_2_ fold change, and the *y* axis shows −log_10_ *P* value. The dashed horizontal line denotes the nominal significance threshold (*P* < 0.01). No fold-change threshold was applied. Genes with *P* < 0.01 are highlighted (red, upregulated in PWH; blue, downregulated in PWH). DGE was assessed using NanoStringDiff with covariate-adjusted negative binomial generalized linear models.

Of the 120 participants in the cohort, 75 participants who provided sufficient SAT for mRNA isolation were included in the analysis; their characteristics are summarized in Supplemental Table 6. Among the 772 fibrosis-related genes analyzed, a total of 43 genes were differentially expressed between PWH and PWoH at *P* < 0.01 after adjustment for age, sex, race, batch, %BF, and prior exposure to legacy d-drugs (Figure 3). Of these, 33 genes were transcriptionally upregulated and 10 were downregulated in PWH. Despite the limitations mentioned above, we also performed a complementary FDR-adjusted analysis (Benjamini-Hochberg) to further support the robustness of our findings and note that the overall pattern of differential gene expression was reassuringly preserved even after this secondary FDR correction (*q* < 0.05), with key fibrosis- and immune-related transcripts maintaining significance (Supplemental Figure 3).

Upregulated transcripts included those encoding key components of the ECM, including collagen type 14 α 1 chain (*COL14A1*), fibril-associated collagen; elastin (*ELN*) which encodes a component of elastic fibers; and laminin subunit γ 1 (*LAMC1*), which encodes the γ-1 subunit of the laminin family of ECM glycoproteins, as well as regulators of ECM remodeling, such as tissue inhibitors of matrix metalloproteinases (*TIMP1* and *TIMP2*). The upregulation of *COL14A1* mRNA levels in the SAT of PWH is particularly noteworthy, as it deviates from the transcriptionally dominant collagens found in the context of obesity-associated SAT fibrosis in PWoH, where types 1, 3, 4, and 6 predominate (27, 34, 46) and in whom collagen type 14 is not well described. This finding suggests that SAT fibrosis in PWH reflects an ECM transcriptional program that is qualitatively different from the canonical program driven by obesity.

In addition to ECM-related genes, several immune signaling transcripts were upregulated in the SAT of PWH in covariate-adjusted differential expression analyses, findings that support immune activation within AT. Given that human AT is identified as a reservoir for HIV, this pattern is consistent with chronic tissue inflammation in this compartment. These upregulated genes included those encoding proteins critical for pathogen recognition and antigen presentation such as *CD14*, a bacterial lipopolysaccharide (LPS) coreceptor expressed on myeloid cells that drives immune activation and inflammation (55), *CD209*, a C-type lectin receptor essential for pathogen binding with polymorphisms associated with HIV-1 susceptibility (56, 57), and *CCL4* (macrophage inflammatory protein 1β), a CCR5 binding chemokine involved in immune cell recruitment and HIV pathogenesis (58, 59). The presence of these immune-related signatures in the SAT underscores that HIV infection is associated with persistent immunologic remodeling that extends beyond classical immune compartments into AT.

By contrast, genes that were transcriptionally downregulated in PWH compared with PWoH included those encoding proteins involved in lipid handling, such as cholesteryl ester transfer protein (*CETP*), fatty acid elongase 6 (*ELOVL6*), fatty acid binding protein 5 (*FABP5*), and patatin-like phospholipase domain-containing 3 (*PNPLA3*), which encodes a triacylglycerol (TG) lipase responsible for TG hydrolysis. This pattern of transcriptional downregulation suggests that the profibrotic and proinflammatory shifts in the SAT of PWH occur at the expense of its lipid metabolic functionality, critical for normal AT health.

### The specific impact of HIV on SAT gene transcription is distinct from that of obesity.

**Figure 4:**
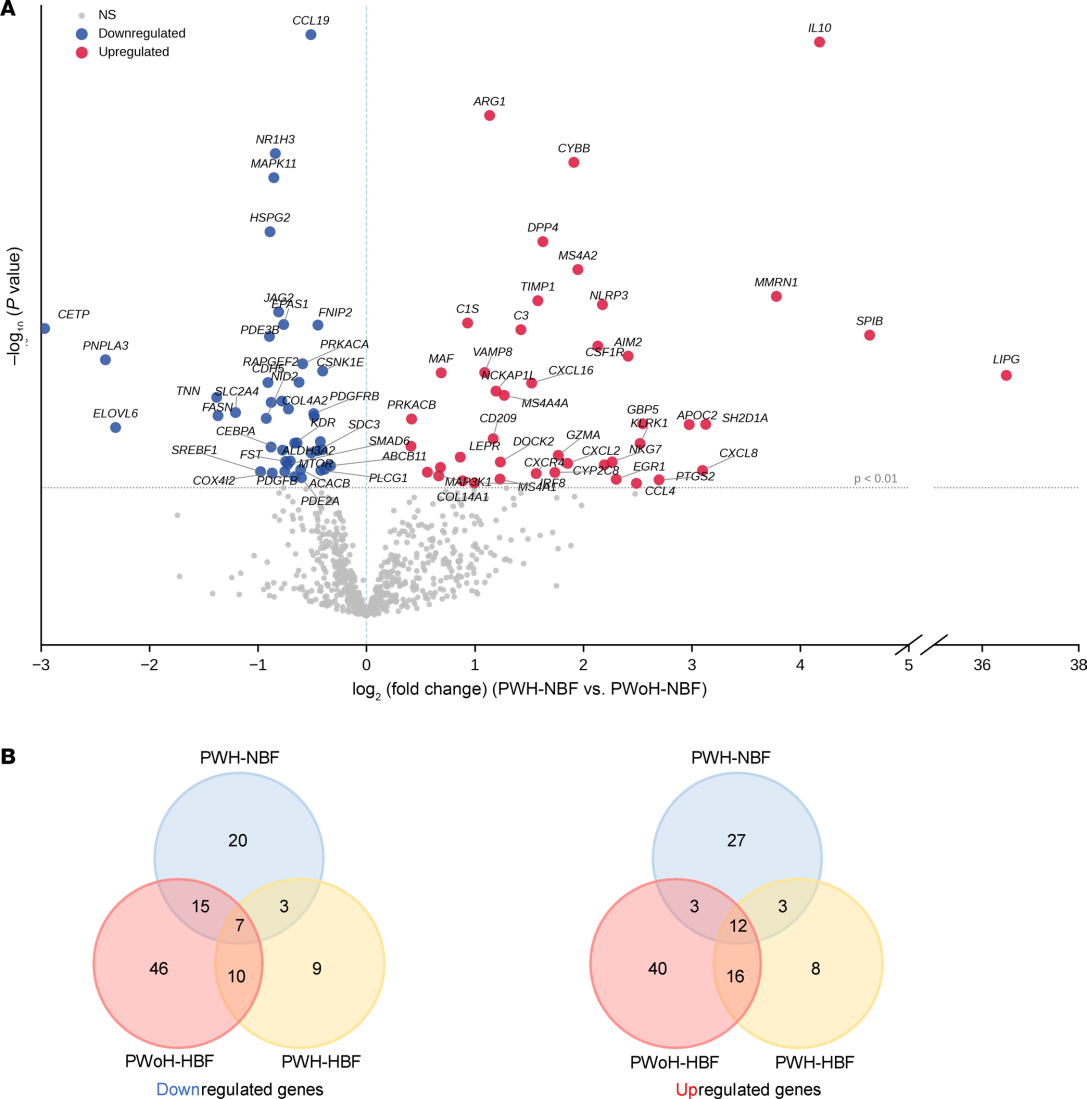
HIV and obesity are associated with distinct patterns of SAT gene transcription. (**A**) Volcano plot of DGE in the SAT of 16 PWH-NBF and 18 PWoH-NBF using the NanoString Human Fibrosis panel. The *x* axis shows log_2_ fold change, and the *y* axis shows −log_10_ *P* value. Genes meeting the nominal significance threshold (*P* < 0.01) are highlighted (red, upregulated compared with PWoH-NBF; blue, downregulated compared with PWoH-NBF). Volcano plots for the remaining pairwise comparisons are in Supplemental Figure 4. (**B**) Venn diagrams showing the number of genes downregulated (left) and upregulated (right) in pairwise comparisons between PWoH-NBF and PWoH-HBF, PWH-NBF, and PWH-HBF, respectively. Overlapping regions indicate genes shared across comparisons, highlighting distinct and convergent transcriptional patterns associated with HIV and increasing adiposity.

Since HIV and obesity have distinct effects on SAT HYP levels, we next sought to formally determine the extent to which the impact of HIV infection on fibrosis-related gene transcription in the SAT is also distinct from that of increasing adiposity in PWoH. We therefore once again stratified our transcriptional dataset into 4 categories: PWoH-NBF, PWoH-HBF, PWH-NBF, and PWH-HBF, mirroring the stratification used in earlier analyses of the relationship between SAT fibrosis and HOMA-IR. This stratification, with participant characteristics summarized in Supplemental Table 7, reduces biological heterogeneity introduced by pooling individuals with varying adiposity, improving the ability to detect context-dependent transcriptional changes. The PWoH-NBF group served as a reference group for each of 3 differential pairwise gene expression (DGE) analyses (Figure 4A and Supplemental Figure 4), the results of which are summarized in a Venn diagram (Figure 4B and Supplemental Table 8). The number of genes downregulated in PWoH-HBF, PWH-NBF, and PWH-HBF, respectively, when compared in a pairwise manner with PWoH-NBF participants is shown, with an analogous approach used to depict upregulated genes for the same pairwise comparisons (Figure 4B). Volcano plots for each pairwise comparison against the PWoH-NBF reference group are presented along with complementary FDR-adjustment in Supplemental Figure 5, with the overall DGE pattern being preserved even after secondary Benjamini-Hochberg correction.

Reassuringly, our DGE analysis recapitulated transcriptional changes that are known to occur in the SAT of people responding to increased adiposity in the general population (51). For example, the comparison of PWoH-HBF versus PWoH-NBF (control) groups revealed several upregulated genes already reported to be induced by obesity, including those encoding inflammatory markers (*CCL13*, *CCL19*) and factors involved in ECM remodeling (*LOX*, *LOXL1*, *LOXL4*, *TIMP2*) (60, 61) (Supplemental Figure 4 and Table 2). Analysis of downregulated genes similarly confirmed well established findings in the literature (61–63), as mRNA levels of genes encoding adiponectin (*ADIPOQ*) and components of the insulin signaling cascade, including the insulin receptor (*INSR*), insulin receptor substrate 1 (*IRS1*), and phosphatidylinositol-4,5-bisphosphate 3-kinase catalytic subunit alpha (*PIK3CA*), were all downregulated in PWoH-HBF (Table 3). These confirmatory findings provide us with confidence in our cohort, tissue collection and processing methods, and analytical pipeline.

### Identifying a fibrosis-related transcriptional signature unique to HIV infection and independent of adiposity.

From these 3 sets of pairwise DGE analyses, we next focused on genes that were differentially regulated at the intersection of 2 or all 3 of the subgroups. Analyzing the DGEs found at these intersections (Figure 4B and Table 2) revealed mRNAs upregulated across each of the 3 DGE analyses we performed versus the reference PWoH-NBF group. Three genes (*COL14A1*, *CSF1R*, and *CYP2C8*) were upregulated in both the PWH-NBF and the PWH-HBF groups versus the PWoH-NBF reference group, indicative of genes that are transcriptionally induced in the SAT of PWH regardless of total body adiposity. By contrast, 3 other genes (*CCL4*, *NCR1*, and *NLRP3*) were transcriptionally upregulated in both the PWH-NBF and PWoH-HBF subgroups versus the reference group, indicative of genes involved both in the response of PWoH to obesity and also in the response of relatively leaner participants to HIV infection. This overlapping set of genes highlights the possibility that some transcriptional components of the SAT fibrosis seen in PWH-NBF reflect responses that are also induced by obesity in the general population. Furthermore, 16 genes were transcriptionally upregulated in the SAT of both PWoH-HBF and PWH-HBF participants compared with the reference group (PWoH-NBF), highlighting genes that are induced by obesity, regardless of HIV infection status.

Our parallel analysis of downregulated genes was also revealing (Figure 4B and Table 3). Specifically, 7 genes were transcriptionally downregulated in all 3 of the pairwise comparisons made versus the PWoH-NBF group. These notably include solute carrier family 2 member 4 (*SLC2A4*), which encodes GLUT4, the glucose transporter responsible for insulin-mediated glucose uptake by ATs, a function that is impaired in the context of IR. Three genes (*FST*, *PNPLA3*, and *TNN*) were transcriptionally downregulated in both PWH-NBF and PWH-HBF participants versus the PWoH-NBF reference group, reflecting transcriptional responses to HIV infection regardless of total body adiposity. Another 10 genes were downregulated in both PWH-HBF and PWoH-HBF groups, highlighting pathways repressed by obesity but independently of the presence or absence of chronic HIV infection. These genes include those encoding proteins associated with intracellular lipid handling (fatty acid binding protein 5; *FABP5*), fatty acid β-oxidation (hydroxyacyl-CoA dehydrogenase; *HADH*), mitochondrial membrane potential regulation (thioredoxin 2; *TXN2*), and mitochondrial respiratory electron transport (ubiquinol-cytochrome c reductase Rieske iron-sulfur polypeptide 1; *UQCRFS1* and ubiquinol-cytochrome c reductase hinge protein; *UQCRH*) that are critical for normal AT metabolic function. In all, our granular subgroup analyses revealed fibrosis-related transcriptional responses in the SAT that are relatively specific to PWH as well as responses that are shared between obesity, regardless of HIV status, and chronically treated HIV infection.

**Figure 5:**
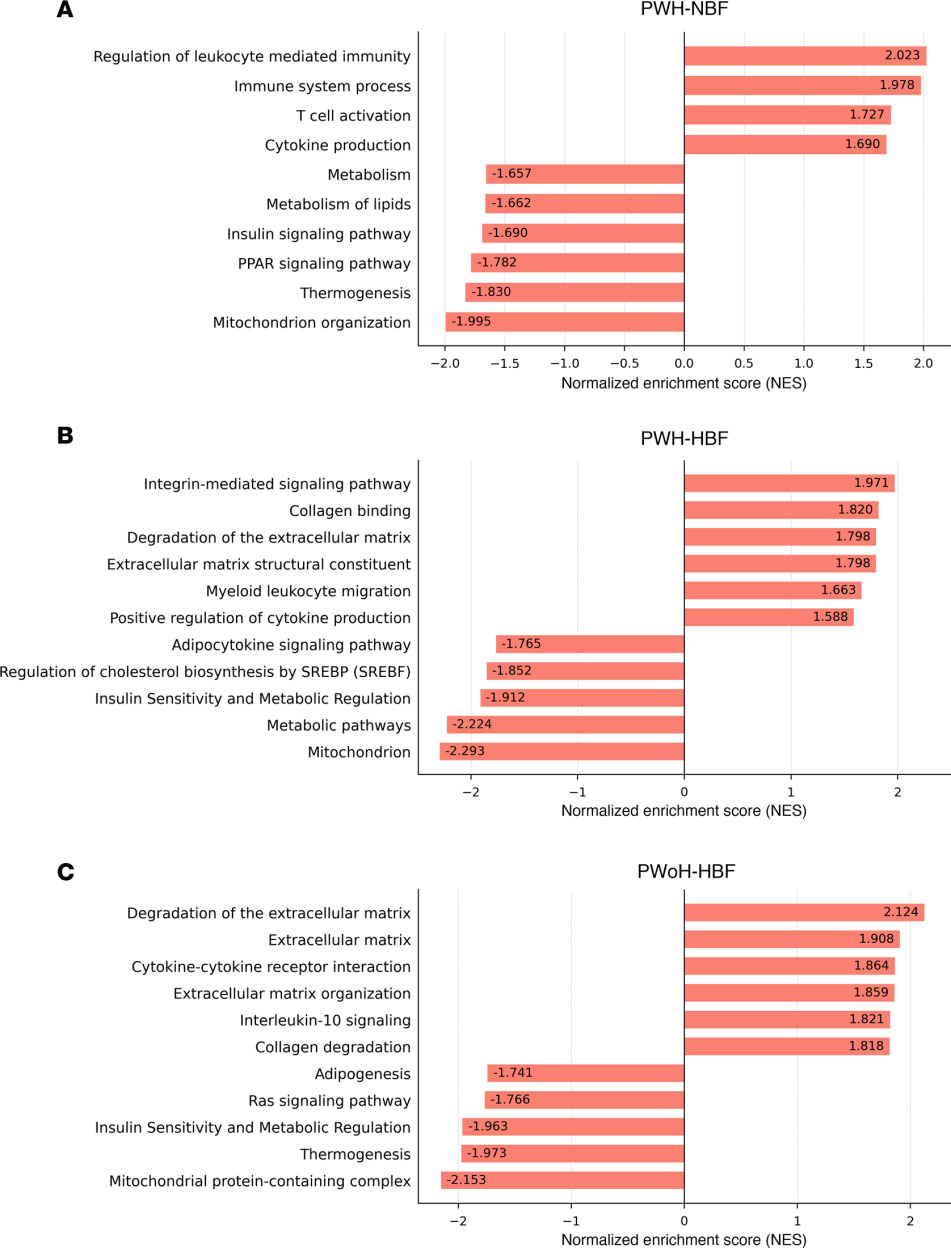
Pathway analysis reveals distinct and shared transcriptional programs across HIV and adiposity subgroups. Normalized enrichment scores (NES) from GSEA for select top significantly enriched pathways in (**A**) PWH-NBF versus PWoH-NBF, (**B**) PWH-HBF versus PWoH-NBF, and (**C**) PWoH-HBF versus PWoH-NBF. Positive NES indicates pathway upregulation, and negative NES indicates pathway downregulation relative to PWoH-NBF. FDR < 0.1. Complete GSEA results, including leading-edge genes, are provided in the Supporting Data Values file.

Functional pathways corresponding to genes differentially expressed in the SAT were identified using Gene Set Enrichment Analysis (GSEA) with curated databases including the Kyoto Encyclopedia of Genes and Genomes (KEGG), Reactome, and Gene Ontology (GO) gene sets. KEGG and Reactome provide pathway-based maps describing molecular interactions and biological processes, while GO offers gene classifications based on molecular function, biological processes, and cellular components. This analysis enabled us to mechanistically distinguish important biological processes associated with excess SAT fibrosis in PWH-NBF from those operational in both PWH-HBF and PWoH-HBF, highlighting potential profibrotic transcriptional mechanisms in PWH that manifest independently of obesity. Figure 5 highlights select top-enriched pathways for each comparison; complete GSEA results, including all significantly enriched pathways and their contributing genes, are provided in the Supporting Data Values file.

In PWH-NBF, pathways related to ECM remodeling, immune activation, and leukocyte migration were significantly upregulated, consistent with a profibrotic and inflammatory SAT phenotype (Figure 5A). Concurrently, there was significant downregulation of pathways related to insulin signaling, thermogenesis, and lipid metabolic processes. Together, these results suggest that SAT dysfunction in PWH-NBF is characterized by immune- and matrix-driven remodeling and impaired energy metabolism, even in the absence of obesity. In PWH-HBF, ECM, and immune-related pathways remained upregulated, overlapping with those upregulated in PWH-NBF, but with the additional enrichment of cytokine signaling, complement activation, and antigen presentation pathways, suggesting that adiposity may further amplify inflammation and immune dysregulation in the setting of HIV (Figure 5B). As expected, the SAT of PWoH-HBF also demonstrated upregulation of ECM pathways and downregulation of thermogenic and adipokine signaling, consistent with features of AT dysfunction that previous studies have established are present in obesity alone (Figure 5C). These findings support the concept that, while obesity independently contributes to fibrotic and metabolic remodeling, chronic, treated HIV introduces additional immune perturbations that distinguish the SAT environment of PWH from that of PWoH, underscoring the potential need for alternative metabolic risk assessment and therapeutic strategies that specifically target ECM remodeling and immune dysregulation in PWH.

### Endotrophin is an adiposity-independent marker of SAT fibrosis in PWH.

We next sought to determine if SAT fibrosis in PWH can be marked in a manner that does not require an invasive tissue biopsy. To this end, we focused on Endotrophin (ETP), a cleavage product of collagen 6A3 that promotes ECM remodeling, immune cell infiltration, and fibrotic signaling in the SAT, contributing to increased tissue stiffness and metabolic dysfunction (26, 64, 65). Beyond its role in AT remodeling, ETP has emerged as a systemic marker of fibroinflammatory burden and metabolic disease severity; circulating ETP levels are strongly associated with IR, progression of chronic kidney and cardiovascular disease, multimorbidity, and increased all-cause mortality across large longitudinal cohorts (66–69). Moreover, there is growing interest in targeting ETP and its downstream signaling pathways to restore metabolic health and glycemic control in metabolically unhealthy obesity (65, 66, 70). However, to our knowledge, ETP levels have not been previously assessed in PWH.

As a foundational step, we examined SAT gene expression from our NanoString dataset, comparing PWH with PWoH. We assessed expression of *COL1A1*, *COL1A2*, *COL3A1*, and *COL6A3*, all of which have been previously associated with AT fibrosis in human obesity. Although differences between groups were not statistically significant, we observed a consistent trend toward higher expression of all 4 genes in PWH (Supplemental Figure 6). These findings suggest that, beyond *COL14A1*, increased fibrotic remodeling in the SAT of PWH may involve multiple collagen genes and offers support for examining ETP as a potential circulating marker of tissue fibrosis in this population.

**Figure 6:**
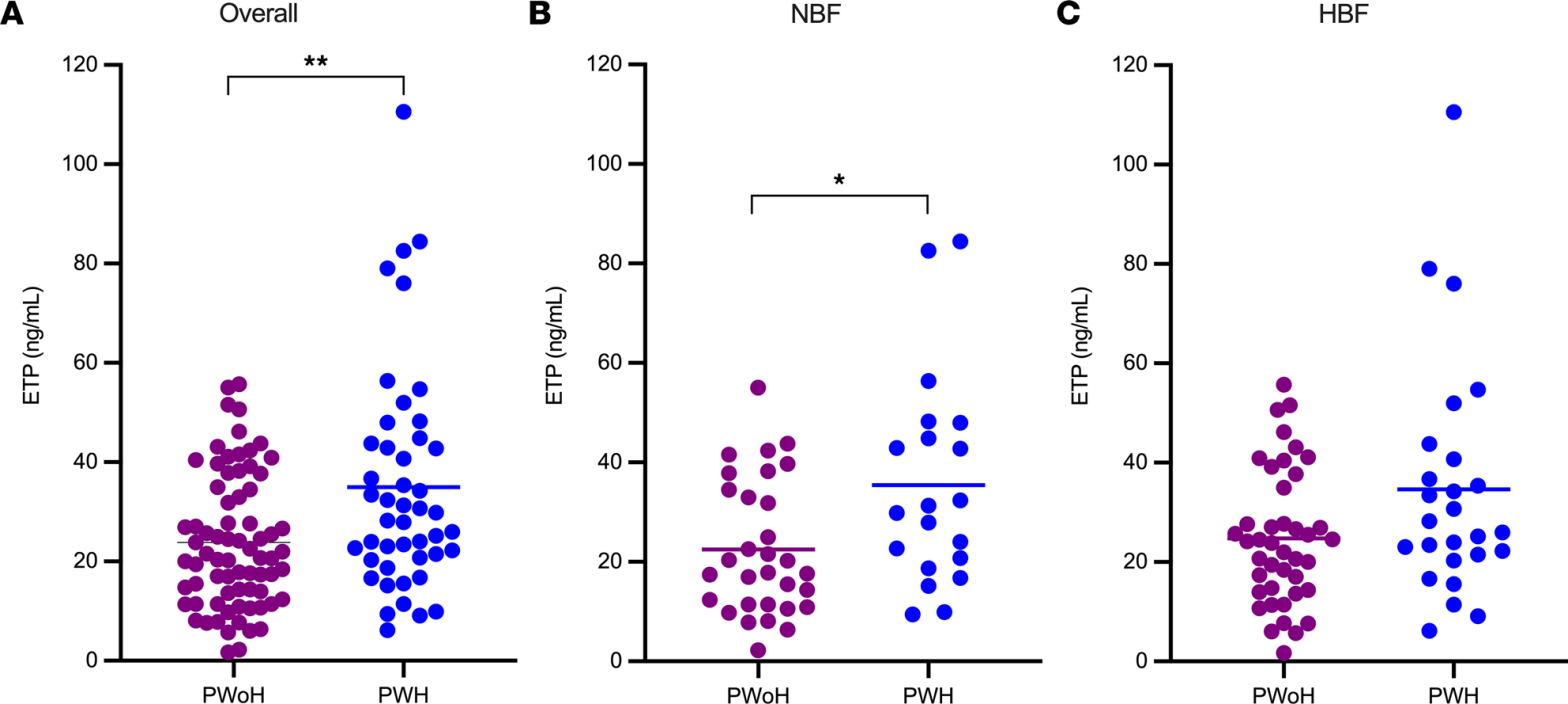
PWH have elevated plasma endotrophin levels, particularly among those with normal body fat. Plasma endotrophin (ETP) concentrations (ng/mL) were compared between persons without HIV (PWoH; purple) and persons with HIV (PWH; blue). (**A**) In the overall cohort (PWoH, *n* = 73; PWH, *n* = 46), plasma ETP levels were significantly higher in PWH than PWoH (*P* = 0.0045). (**B**) Among participants with normal body fat (NBF; PWoH, *n* = 31; PWH, *n* = 20), plasma ETP levels were significantly elevated in PWH compared with PWoH (*P* = 0.018). (**C**) Among participants with higher body fat (HBF; PWoH, *n* = 42; PWH, *n* = 26), the difference did not reach statistical significance (*P* = 0.10). Groups were compared using the Mann-Whitney *U* test. *P < 0.05, **P < 0.01.

Interestingly, PWH had higher plasma ETP levels than PWoH in unadjusted analyses (Figure 6A; *P* = 0.0045), and this remained highly significant after multivariable linear regression adjustment for sex, age, race, and %BF (Supplemental Table 9 and Supplemental Figure 7A). Moreover, circulating ETP correlated positively with HOMA-IR in both PWH (Spearman ρ = 0.42, *P* = 0.006) and PWoH (ρ = 0.28, *P* = 0.019) even after adjusting for age, sex, race and ethnicity. Notably, neither %BF, sex, age, nor race were independently associated with circulating ETP levels in this model, indicating that the ETP elevation in PWH is not explained by differences in body composition or demographic factors. In a multivariable model restricted to PWH (Supplemental Table 10, Model 1), neither legacy d-drug exposure, current INSTI use, CD4^+^ T cell count, nor %BF was associated with plasma ETP levels. To evaluate robustness, we sequentially adjusted for additional demographic covariates: sex (Model 2), age (Model 3), and race (Model 4). Effect estimates for the primary covariates remained stable across all models, and no additional covariate reached statistical significance.

We next again performed adiposity-stratified subgroup analyses. Among individuals with NBF, plasma ETP levels were significantly higher in PWH than PWoH in both unadjusted (*P* = 0.018; Figure 6B) and adjusted analyses (Supplemental Figure 7B), and %BF was not associated with plasma ETP in this subgroup (Supplemental Table 11). Among individuals with HBF, the difference between PWH and PWoH did not reach statistical significance in unadjusted (*P* = 0.10; Figure 6C) or adjusted analyses (Supplemental Figure 7C), although %BF was a significant independent predictor of circulating ETP levels in this subgroup (Supplemental Table 11). These analyses identify plasma ETP as a noninvasive marker of AT remodeling that is elevated in PWH independently of ART history and demographic factors. This elevation was significant among individuals with NBF, where %BF did not influence ETP levels, and trended in the same direction among those with HBF, where adiposity itself, rather than HIV status, was the dominant predictor of circulating ETP levels. As with tissue HYP, the HIV-associated elevation in ETP was most evident among individuals with relatively normal adiposity, reinforcing that the link between HIV and fibrotic remodeling goes beyond what can be attributed to excess body fat.

## Discussion

In this study, we demonstrate that PWH have increased SAT fibrosis compared with PWoH, and that it occurs in a manner uncoupled from obesity. In PWH-NBF, SAT fibrosis is strongly associated with IR, whereas commonly used anthropometric measures (BMI, %BF) are not similarly predictive. These findings position SAT fibrosis as a key correlate of metabolic dysfunction in PWH, independent of obesity, and emphasize the need for alternative metrics to assess metabolic risk in this population.

Current strategies to mitigate the increased risk of T2D in PWH focus primarily on routine screening and lifestyle interventions, similar to those applied in PWoH. The American Diabetes Association guidelines recommend screening prior to initiating ART, followed by annual follow ups to facilitate early diagnosis. However, the mechanisms underlying this heightened risk remain unclear. Our data highlight the limitations of relying on BMI as a metric for assessing metabolic risk in PWH, particularly in nonobese individuals, and underscore instead the potential importance of developing mechanistically tailored risk assessment tools and even targeted therapies to mitigate T2D risk in PWH.

Few studies have previously explored the role of SAT fibrosis in metabolic health among PWH. Bailin et al. (71) reported transcriptional and cellular changes in the SAT from a cohort of PWH with varying T2D status, including individuals without diabetes, with prediabetes, and with T2D implicating pathologic fibroblasts in the accrual of VAT and the development of T2D. However, the absence of a PWoH comparator group limited the ability to distinguish HIV-specific effects from those attributable to obesity or diabetes more broadly. By contrast, our study directly compares PWH with PWoH and includes stratification by adiposity, enabling separation of HIV-associated transcriptional changes from those driven by excess fat mass alone. Importantly, our measurements of SAT HYP content confirm that the transcriptional upregulation of ECM genes in PWH translate into actual protein-level changes, strengthening the link between chronic HIV and SAT fibrosis. Further, by showing that our findings stand up in the face of adjustment for exposure to legacy d-drugs and current INSTI usage, which are known to induce mitochondrial toxicity and durable alterations in adipocyte function and AT remodeling, we conclude that the increased SAT fibrosis seen in PWH is not simply explained by the residual effects of ART-related toxicity or by INSTI-dependent metabolic alterations.

Our findings align with prior research showing the association between SAT fibrosis and metabolic dysfunction in the general population (24, 27, 34), in which collagens type 1, 3, 4, and 6 dominate in pathological AT and contribute to AT dysfunction (27, 34, 46). However, whereas there was indeed a trend toward upregulation of these collagen genes in PWH, we identified *COL14A1* as being uniquely upregulated in PWH, independent of excess adiposity. Collagen type 14 (COL14), a fibril-associated collagen with interrupted triple helices, consists of 2 collagenous domains that are important for the formation of the COL14 triple helical structures and for the binding to other fibrillar collagens and 3 noncollagenous domains (72, 73).

COL14 is implicated in cell proliferation, differentiation, and fibrillogenesis and it has been identified in various tissues, including skin, tendon, cornea, and lung, where it localizes near blood vessels, airway smooth muscle, and bronchial epithelium (74–76). While COL14 function in ECM remodeling and mechanical stress regulation is well-established in these tissues, very few studies have reported on the contribution of *COL14A1* in AT, and its contribution to AT structure, fibrosis, and metabolic dysfunction remains largely uncharacterized. In our study, *COL14A1* was upregulated in PWH compared with PWoH, suggesting that COL14 may have a distinct role in SAT remodeling in the context of HIV. A microarray analysis previously found that *COL14A1* mRNA levels decrease during the adipogenic differentiation of human mesenchymal stem cells (77), whereas in 3T3-L1 cells, the FN type III domain of Col14 was shown to promote adipogenesis (78). A recent study found that *COL14A1* mRNA expression was elevated in PWH with increased VAT volume (71), an interesting corroboration of our findings. Together, these data suggest that *COL14A1* may contribute to fibrotic remodeling particularly in the SAT of PWH, where fibrosis is associated with IR, a key determinant of T2D risk.

Genes related to cellular processes known to promote tissue fibrosis in general were also transcriptionally upregulated in the SAT of PWH versus PWoH. For example, *TIMP1* levels were also shown to be induced in in vitro models of human metabolic dysfunction associated steatohepatitis–associated (MASH-associated) liver fibrosis (79). Our analysis also showed upregulation of the gene encoding Janus kinase 2 (*JAK2*), a kinase reported to mediate IL-5Rα–dependent profibrotic signaling in the setting of lung fibrosis (80). Importantly, JAK2 signaling is also important in response to inflammatory cytokines known to be elevated in HIV including FN-γ and IL-6. Collectively, these findings suggest that chronic HIV infection transcriptionally induces pathways in the SAT known to mediate fibrosis in multiple tissue environments.

Additionally, our transcriptional analysis revealed overlapping pathways between HIV infection and obesity. For example, *CCL4* and *NLRP3* were upregulated in both PWH and obese individuals both with and without HIV infection. *CCL4* plays a dual role in HIV suppression and metabolic inflammation, while *NLRP3* is a key component of the NLRP3 inflammasome implicated in both obesity and HIV-related tissue inflammation (81–83). Such transcriptional overlap warrants a dedicated search for cellular origins and functional impacts, which could yield mechanisms contributing to IR and T2D risk shared between chronic HIV and other pathological contexts.

Clinically, the pathways identified here, particularly those related to impaired adipogenesis and excessive ECM remodeling, underscore the potential value of moving beyond weight loss–focused strategies to consider interventions that target AT structure and function. Our findings support the concept that SAT fibrosis contributes to metabolic risk independent of adiposity, especially in PWH. This points to a potential role for therapeutic strategies aimed at improving AT expandability and reducing fibrotic remodeling. Antifibrotic agents currently in development for conditions such as liver fibrosis and MASH, including inhibitors of TGF-β signaling or LOXL2, may eventually have relevance in the context of AT fibrosis. Further research is needed to evaluate whether such therapies could benefit metabolic health in PWH by mitigating fibrosis-driven IR.

Building on these findings, we focused on ETP, a circulating cleavage product of COL6A3 that has been associated with tissue remodeling, fibrosis, and metabolic dysfunction. ETP was shown to promote inflammation and insulin resistance in ATs, where its levels increase with obesity and associated AT fibrosis (26). Human studies have also revealed a significant upregulation of ETP in the AT of individuals with obesity and T2D (84, 85). Indeed, ETP is also increasingly being recognized as a key mediator of inflammation and fibrosis in the pathophysiological remodeling seen in both metabolic and other pro-fibrotic diseases (86). ETP is highly expressed in tumors and accelerates cancer progression, including breast and liver cancer (87, 88). Clinically, elevated circulating ETP levels are associated with poor outcomes in chronic kidney disease (68) and chronic liver disease (69) and have been identified as predictive of the response to insulin-sensitizing therapies (84).

More recently, circulating ETP levels were strongly correlated with IR in individuals with obesity and with both chronic multimorbidity and all-cause mortality in large population-based cohorts, supporting its role as an integrative marker of systemic fibroinflammatory and metabolic disease burden (66, 67). Consistent with these reports, plasma ETP levels in our cohort also correlated with HOMA-IR, further supporting its relevance as a marker of metabolic dysfunction. Importantly, our findings extend this framework beyond obesity-associated fibrosis to include PWH even in the absence of increased adiposity. The higher plasma ETP levels observed in PWH, particularly in PWH-NBF, supports a model in which HIV-related factors may contribute to AT remodeling, independent of excess fat mass.

A notable distinction emerged between ETP and tissue HYP with respect to adiposity: whereas %BF was a significant independent predictor of SAT HYP in the overall model, consistent with bulk collagen deposition that accompanies fat mass expansion, %BF was not associated with circulating ETP. This dissociation suggests that tissue HYP and circulating ETP capture fundamentally different facets of ECM biology; HYP reflects total collagen content that scales with adiposity, whereas ETP may more specifically mark active fibrotic signaling processes that are upregulated in PWH regardless of body fat levels. Given that ETP circulates and is detectable from a simple blood draw, it holds potential as a tractable noninvasive biomarker for SAT fibrosis and its associated metabolic consequences in PWH. Future studies should explore whether targeting ETP-mediated fibrosis could mitigate metabolic dysfunction in HIV, providing a potential therapeutic avenue for this high-risk group.

Despite its strengths, our study also has limitations. Although the study cohorts were well matched for age, some differences in sex and race and ethnicity remained. Specifically, it was challenging to recruit women with HIV despite targeted outreach, resulting in a PWH group that included a higher proportion of men and a greater proportion of Black participants. These demographic patterns are consistent with the real-world epidemiology of HIV in the United States, where Black men bear a disproportionate burden of disease and its associated metabolic complications, which may enhance the generalizability of our findings to the populations most affected. To account for these imbalances, all multivariable analyses were adjusted for age, sex, race, and percentage body fat. As with any cross-sectional study, residual confounding cannot be fully excluded, and we cannot rule out the possibility that an unidentified factor associated with HIV independently contributes to both SAT fibrosis and insulin resistance. However, HIV status remained a significant independent predictor of SAT fibrosis across all adjusted models, supporting the robustness of this association.

Our transcriptomic analysis was limited to a targeted fibrosis gene panel, which may not encompass all pathways relevant to AT dysfunction in PWH. Nevertheless, this focused design allowed for sensitive detection of coordinated transcriptional changes within fibrosis-related pathways that are directly aligned with our biochemical measures of SAT fibrosis. Given the specific and hypothesis-driven composition of this targeted fibrosis panel, stringent multiple-testing correction may obscure biologically relevant transcriptional patterns within predefined pathways. Although we performed complementary FDR-adjusted analyses, conservative correction strategies are known to reduce sensitivity in this setting (66, 67). Future studies employing genome-wide transcriptomic approaches with minimal bias as well as independent validation cohorts will be necessary to further define the biological significance of our findings. Moreover, because we assessed bulk transcriptomics, we cannot differentiate altered gene expression within a given cell type from a difference in the admixture of cells with different transcriptional profiles. For example, reduced lipid metabolism gene expression in PWH may reflect an increased relative abundance of immune cells in the AT and a reduced abundance of adipocytes rather than a direct effect on adipocyte function. Future studies employing single-cell transcriptomic or multi-omic approaches will be important for resolving cell-type–specific contributions to SAT fibrosis and metabolic dysfunction in PWH.

Our analyses accounted for both prior exposure to legacy ARTs, including thymidine analogs and d-drugs, as wells as ongoing INSTI use, and we did not find evidence that ART class independently explained excess SAT fibrosis in the PWH we examined. Nonetheless, the relatively small sample size and the high prevalence of INSTI-based regimens in this cohort limit our ability to fully disentangle the effects of HIV infection itself from potential ART-related influences. Similarly, our study did not directly assess HIV-encoded viral proteins; future studies could examine whether specific viral proteins contribute to fibrotic pathways in adipose stromal cells in vitro, which would complement the findings of the present work. These findings underscore the need for larger, mechanistic studies to more precisely define the relative contributions of viral persistence, immune activation, and ART to AT remodeling in PWH.

Finally, the cross-sectional nature of this study also precludes causal inferences. Longitudinal studies will be essential to clarify temporal relationships and to determine whether SAT fibrosis and plasma ETP levels predict long-term metabolic outcomes in PWH. Future research should also prioritize the recruitment of underrepresented groups, particularly women with HIV, to improve demographic matching and reduce potential confounding in comparative analyses.

In conclusion, our findings reveal the presence of increased SAT fibrosis in PWH, particularly in nonobese participants, as well as its strong association with IR, independent of obesity, in this population. The distinct transcriptional profile of SAT fibrosis in PWH, including the upregulation of *COL14A1*, highlights unique HIV-driven mechanisms associated with AT dysfunction. Elevated plasma ETP levels in PWH support the use of ETP as both a potential biomarker to track SAT fibrosis and a plausible future therapeutic target. Our findings challenge the reliance on traditional anthropometric measures for assessing metabolic risk in PWH and instead provide a foundation for future studies to elucidate the cellular and molecular drivers of HIV-associated SAT fibrosis and its metabolic consequences.

## Methods

### Sex as a biological variable.

Our study examined male and female participants. Sex differences are reported and were considered as a biological variable.

### Participants and enrollment procedures.

We enrolled people with and without HIV aged 18–75 years from 2 UCSF-based cohorts: the SCOPE cohort and the Inflammation, Diabetes, Ethnicity, and Obesity (IDEO) cohort. The SCOPE cohort is a longitudinal study of PWH and PWoH, focusing on detailed clinical and virologic outcomes. For this study, we included SCOPE participants who had been on ART for at least one year and maintained viral suppression with undetectable plasma HIV RNA levels for at least 12 months. The IDEO cohort comprises people without HIV recruited from UCSF and Zuckerberg San Francisco General Hospital clinics, as well as through local public advertisements. Established members of the IDEO cohort who met study criteria were included. Exclusion criteria for both cohorts included a prior diagnosis of T2D (HbA1c > 6.5% or a physician diagnosis with diabetes medication use), use of anti-inflammatory medications or glucocorticoids, a history of organ failure, autoimmune disorders, cancer, or substantial weight change (> 5%) in the preceding three months. Race and ethnicity were self-identified by participants at study enrollment using categories defined by the investigators in accordance with NIH/HHS standards. We stratified our sampling by HbA1c to ensure a comparable distribution of participants with prediabetes in each group, allowing us to assess whether the associations between AT and IR differed in the context of HIV status.

### RNA isolation and transcriptional analysis.

Total RNA was extracted from 70 to 100 mg of whole SAT using the RNeasy Lipid Mini Kit (QIAGEN) following the manufacturer’s protocol. RNA concentration (ng/μL) and purity (A260/280) were determined for each RNA sample using the Nanodrop 1000 (ThermoFisher Scientific). Individual mRNA levels and the transcriptional patterns for an array of fibrosis-associated genes in the SAT were analyzed using the NanoString customized fibrosis transcriptomic panel, consisting of 14 probes for quality control (6 positive controls, 8 negative controls), 8 probes for predetermined housekeeping genes (*ACAD9*, *ARMH3*, *CNOT10*, *GUSB*, *MTMR14*, *NOL7*, *NUBP1*, and *HPRT1*), and 772 probes for endogenous genes known to be involved in tissue fibrosis and the ECM. Raw counts were first filtered through a quality control step utilizing the 14 synthetic probes, then normalized to the mRNA levels of the housekeeping genes. The R package NanoStringDiff was used for differential gene expression (DGE) analysis of the normalized counts. DGE analysis between PWH and PWoH was adjusted for age, sex, race, ethnicity, %BF and batch.

### Statistics.

Statistical analyses were carried out in Python, except for those pertaining to SAT gene expression analysis, as described above. To compare the basic characteristics of PWH with PWoH, categorical variables were analyzed using the χ^2^ test. Continuous variables were analyzed using Student’s *t* test for normally distributed data and the Mann-Whitney *U* test for nonnormally distributed data. To separate the effects of HIV status from those of adiposity, participants were categorized into 4 groups based on HIV status and percent body fat (%BF). Sex-specific %BF cutoffs were used to define adiposity, with thresholds of 25% for males and 35% for females to classify participants as having normal or high %BF. Participants were therefore classified as PWoH with normal %BF (PWoH-NBF), PWoH with high %BF (PWoH-HBF), PWH with normal %BF (PWH-NBF), or PWH with high %BF (PWH-HBF). Partial correlation analysis was carried out to understand the degree of linear association between each pair of phenotypic variables in our data. Given the small number of participants in individual race categories other than Black and White, race was collapsed into 3 categories: Black, White, and Other (Asian, Pacific Islander, more than one race, and unknown), for all adjusted analyses. Spearman partial correlation coefficients were computed using the pingouin package, adjusting for sex, age, race, and d-drug exposure; pairs with *P* < 0.05 were annotated on the resulting heatmap.

Multivariate regression analysis was performed to evaluate the effects of age, sex, race, %BF, INSTI use, legacy d-drugs, and HIV status on SAT HYP and plasma ETP levels. Records with missing covariates were excluded; continuous outcomes were Box-Cox power transformed; and Ordinary Least Squares (OLS) models were fit using the stats package with checks for linearity, residual normality, and homoscedasticity. Adjusted effects of HIV on HYP and ETP were predicted using the mean for each continuous covariate (age, %BF) and the mode for each categorical covariate (race, sex) and displayed as adjusted dot plots with 95% confidence interval error bars.

For NanoString-based DGE analysis, we followed the manufacturer’s recommendations and did not apply a genome-wide multiple-testing correction; instead, a stringent *P* < 0.01 threshold to identify differential expressed genes. The 772-gene NanoString Human Fibrosis panel is hypothesis-driven, with all targets preselected for known or suspected involvement in tissue fibrosis., such that global FRD adjustments would be overly conservative and inflate Type II error (false negatives), a concern echoed in NanoString’s analysis guidelines (54) and prior studies using similar panels (48, 50–53). As a complementary approach, Benjamini-Hochberg false discovery rate (FDR) correction was applied secondarily, with q values reported alongside nominal *P* values. No fold-change threshold was imposed, to avoid modest but potentially biologically meaningful effects. To distinguish HIV-associated from adiposity-associated transcriptional pattern, 3 pairwise, DGE analyses (PWoH-HBF, PWH-NBF and PWH-HBF versus the PWoH-NBF reference group) were performed using the R package NanoStringDiff adjusted for age, sex, race/ethnicity, and batch.

Pathway analysis was performed using gene set enrichment analysis (GSEA) with the FGSEA algorithm, implemented via the clusterProfiler and ReactomePA packages in the R programming language. First, each gene marker was assigned a score based on the fold-change and *P* value from the differential gene expression analysis. The calculated scores were then used to create a ranked gene list, followed by mapping the gene identifiers to their corresponding ENTREZ IDs. The ranked list was then used to assess pathway enrichment across multiple databases (GO, KEGG, and REACTOME). Significantly enriched pathways were identified using a *P* value cutoff (*P* < 0.1) after controlling for multiple hypothesis testing using false discovery rate adjustment.

Additional methodological details are provided in the Supplemental Materials.

### Study approval.

All participants provided informed consent, and the study received approval from the University of California San Francisco (UCSF) Institutional Review Board (14-14128).

### Data availability.

Data values underlying all figures and tables are provided in the accompanying Supporting Data Values file. Raw NanoString count data are available at FigShare at https://doi.org/10.6084/m9.figshare.28971413.v1

## Author contributions

DLA, SKK, and PWH conceptualized and designed the study. AER, ME, TRF, and JGV assisted with participant enrollment, coordinated research visits, collected biospecimens, and entered patient metadata into the study database. SMBM, TATP, DIB, and DLA processed blood and tissue samples and performed experiments. NZ and ZA developed the antibodies and the ETP ELISA assay. DB performed the ETP assay. MKC and AA cleaned the data, performed analyses, and contributed to figure design, which was supervised by DLA. SGD, and PES. SGD and PES provided resources and revised the manuscript for important intellectual content. DLA and MKC wrote the original draft. PWH, SKK, and DLA contributed to the review and editing of the manuscript. All authors reviewed and approved the final version of the manuscript.

## Conflict of interest

The authors have declared that no conflict of interest exists.

## Funding support

This work is the result of NIH funding, in whole or in part, and is subject to the NIH Public Access Policy. Through acceptance of this federal funding, the NIH has been given a right to make the work publicly available in PubMed Central.

- National Institutes of Health, R01DK141041 (PWH, SKK).
- National Institutes of Health, R01DK112304 (PWH, SKK).
- National Institutes of Health, R56DK133997 (PWH, SKK).
- National Institutes of Health, K08DK124679 (DLA).
- National Institutes of Health, T32DK007418 (MKC).
- National Institutes of Health, P30DK098722 (UCSF Nutrition Obesity Research Center).
- National Institutes of Health, P30AI027763 (UCSF-Bay Area Center for AIDS Research).
- Robert Wood Johnson Foundation Harold Amos Medical Faculty Development Program (DLA).
- Harold Amos Medical Faculty Development Program (DLA).

## Supplementary Material

Supplemental data

ICMJE disclosure forms

Supporting data values

## Figures and Tables

**Table 1:**
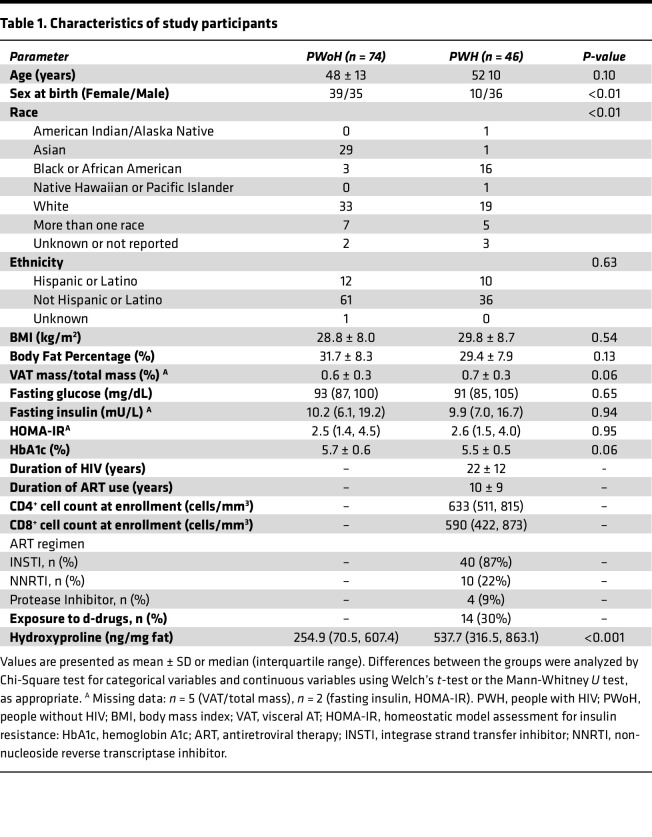
Characteristics of study participants

**Table 2:**
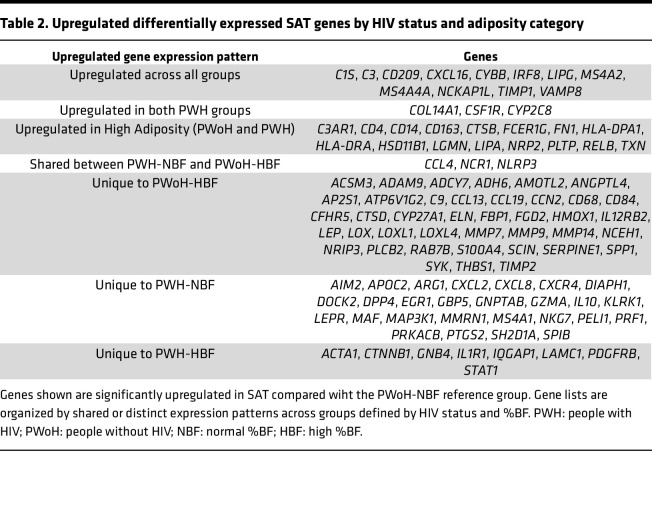
Upregulated differentially expressed SAT genes by HIV status and adiposity category

**Table 3:**
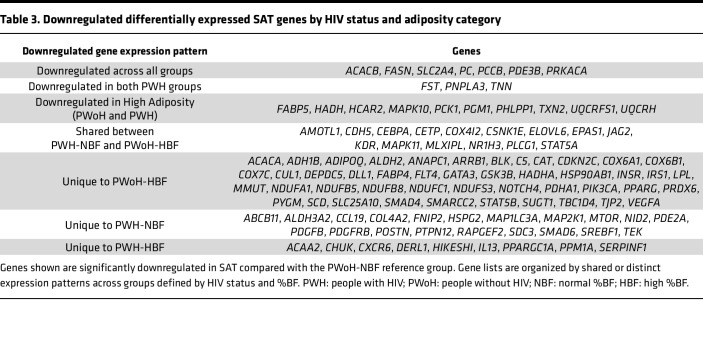
Downregulated differentially expressed SAT genes by HIV status and adiposity category

## Version 1 06/30/2026

In-Press Preview

## Version 2 08/10/2026

Electronic publication
