# Supplementary material for: A distinct form of fat fibrosis is linked to insulin resistance in people with HIV
Source: JCI Insight. 2026 Jun 30;11(15):e195922. doi: 10.1172/jci.insight.195922 (PMC13463614; doi:10.1172/jci.insight.195922)
Supplement: Supplementary file 1 [file jciinsight-11-195922-s225.pdf]

Supplemental Materials for

A Distinct Form of Fat Fibrosis is Linked to Insulin Resistance in People with HIV

Diana L. Alba, Alaa Abdellatif, Moon K. Choi, Stephen Brown Mayfield, Thuy An T. Pham, David I. Berrios, Antonio E. Rodriguez, Marin Ewing, Tony Figueroa, Judy Gonzalez-Vargas, Ningyan Zhang, Zhiqiang An, Dawei Bu, Steven G. Deeks, Philipp E. Scherer, Peter W. Hunt, Suneil K. Koliwad

Corresponding authors: Diana L Alba diana.alba@ucsf.edu

Suneil K. Koliwad: Suneil.koliwad@ucsf.edu

The PDF file includes Supplemental Methods, Supplemental Figures 1-7 and Supplemental Tables 1-11. Other Supplementary Material for this manuscript includes the following: Supporting Data Values file

Supplemental Methods

Body composition and anthropometric measurements. Participants' height and weight were measured using a standard stadiometer and scale, with BMI (kg/m2) calculated from two averaged measurements. Body composition was estimated by DXA using a Hologic Horizon/A scanner (3-minute whole-body scan, <0.1 mGy) per manufacturer protocol. This device accurately measures participants up to 450 lbs. and employs high-performance and “offset” scanning techniques on those whose bodies are wider than the table. Standard DXA readouts include total percentage body fat (%BF), fat mass, lean mass, total body mass and VAT volume. A single technologist analyzed all DXA measurements using Hologic Apex software (13.6.0.4:3) following International Society for Clinical Densitometry guidelines. VAT was measured in a 5-cm-wide region across the abdominal region above the iliac crest, coincident with the fourth lumbar vertebrae, matching the region used for computed tomography (CT)-based VAT analysis (1), and approached validated against CT (r^2: 0.949-0.959) (2).

AT sample collection. SAT samples were collected from most participants after a 10-hour fast via aspiration biopsy under local anesthesia using 2% lidocaine. A 2.1 mm blunt-sided, ported liposuction catheter (Tulip CellFriendly™ GEMS Miller Harvester, Tulip Medical Products, San Diego, CA) was used to collect tissue from approximately 5 cm lateral to the umbilicus. In some participants, SAT was instead collected intraoperatively during bariatric surgery. After removal of visible connective tissue, blood, and clots, samples were washed with Krebs-Ringer bicarbonate buffer containing 1% BSA and immediately frozen in liquid nitrogen.

Collagen content measurement. The collagen content of SAT was measured by determining tissue HYP levels using the Quick Zyme Sensitive Tissue Hydroxyproline Assay (QuickZyme Biosciences, the Netherlands) according to the manufacturer’s instructions. Briefly, SAT samples were acid hydrolyzed using 6M HCl at 95°C for 20 hours. The HYP content was then assessed by spectrophotometry at 570nm.

ELISA measurements of circulating ETP. Plasma samples were freshly prepared from blood samples collected after a 10 hour fast and preserved at –80°C until analysis. To quantitatively determine the levels of circulating ETP, 96-well plates (Corning Costar) were coated with rabbit monoclonal anti-endotrophin antibodies prepared in house at 2 µg/mL concentration. Plasma samples were titrated at a series of dilutions in 1X PBS, then added to an anti-endotrophin coated plate. A high affinity specific anti-endotrophin antibody (ETN-1Rb) was utilized as primary, with an anti-rabbit Fab2-HRP antibody (111-035-006, Jackson ImmunoResearch) used as a secondary antibody to amplify the ETP signal, at dilutions suggested by the manufacturer. The coating antibody and the primary detection antibody (ETN-1Rb) were both generated in-house in the laboratory of N. Zhang and Z. An (Texas Therapeutics Institute, Brown Foundation Institute of Molecular Medicine, University of Texas Health Science Center at Houston, Houston, TX). A purified recombinant ETP protein was titrated across a series of concentrations (0–50 ng/mL) to establish a standard curve for calculating plasma ETP levels.

Supplemental Figures

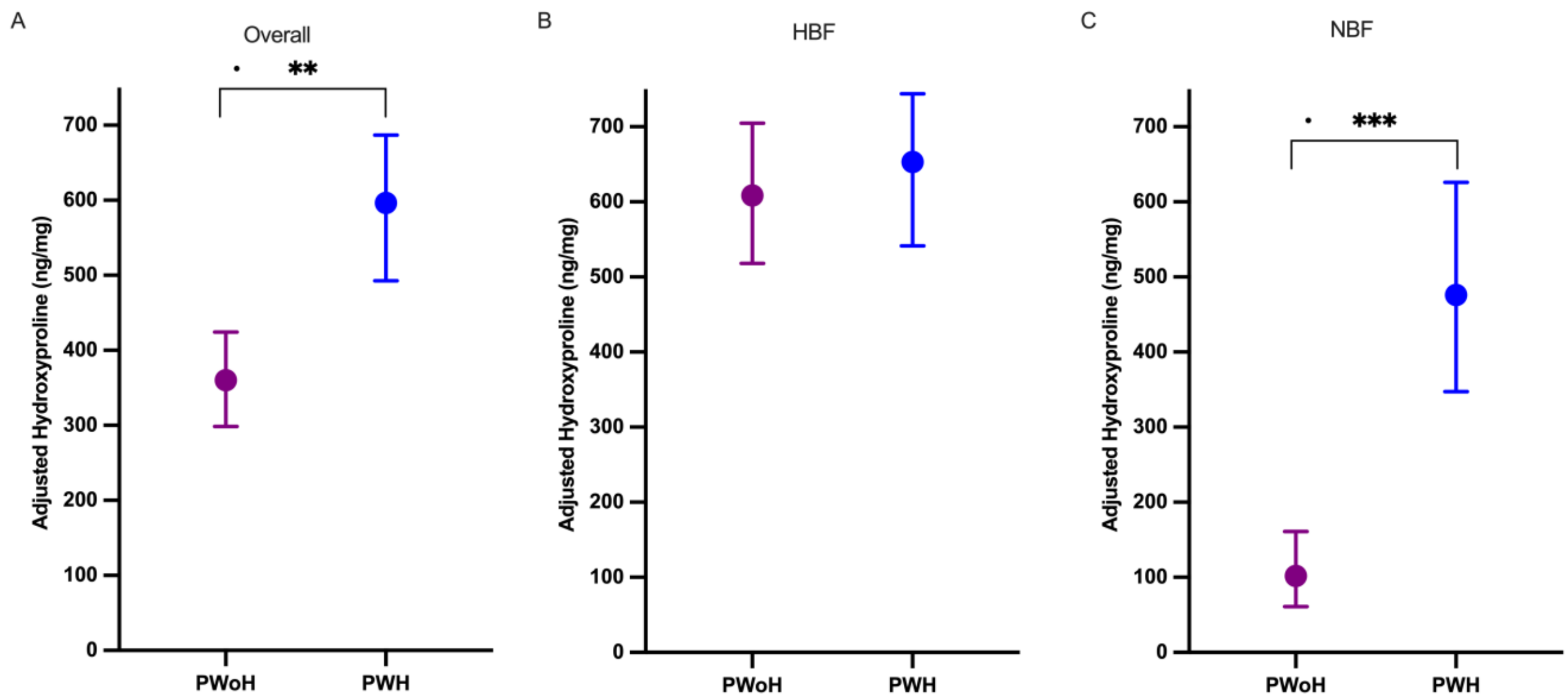

Supplemental Figure 1. Shown are model-derived adjusted estimates of SAT hydroxyproline (HYP) levels in people with HIV (PWH) and people without HIV (PWoH). HYP levels were estimated using multivariable linear regression models with HIV status as the primary exposure, adjusting for sex, age, and race/ethnicity, and analyses were stratified by adiposity category as defined in the Methods. **(A)** In the full cohort, PWH exhibited higher adjus ted SAT HYP levels compared with PWoH (PWoH n = 74, PWH n = 46). **(B)** Among participants with high adiposity (%BF ≥ 25% in males and ≥ 35% in females), adjusted SAT HYP levels did not differ between PWH and PWoH (PWoH n = 43, PWH n = 26). **(C)** Among participants with normal adiposity (% BF < 25% in males and < 35% in females), adjusted SAT HYP levels were significantly higher in PWH compared with PWoH (PWoH n = 31, PWH n = 20). Adjusted values shown are derived from the fitted regression models; error bars represent 95% confidence intervals. P-values correspond to the regression coefficient for HIV status within each model and were calculated from the t-statistic using appropriate degrees of freedom. **p < 0.01; ***p < 0.001.

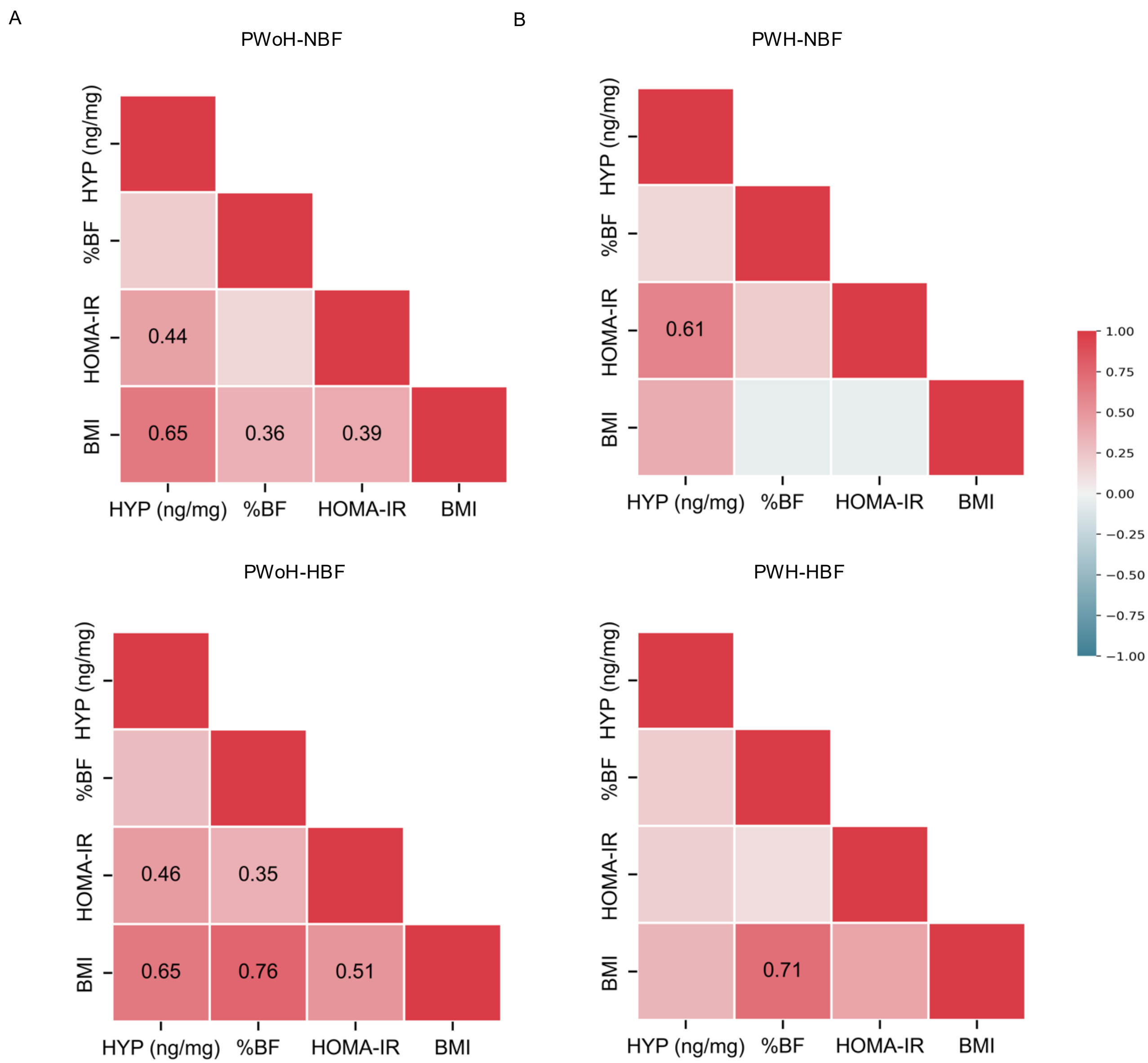

Supplemental Figure 2. Partial Spearman correlation analyses were performed to evaluate relationships among subcutaneous adipose tissue hydroxyproline (HYP), insulin resistance (HOMA-IR), BMI and whole-body percent fat (%BF) in PWoH and PWH, further grouped by normal adiposity and high adiposity as previously defined. **(A)** Correlation matrices for PWoH with normal adiposity (NBF, n = 30) and high adiposity (HBF, n = 43). **(B)** Correlation matrices for PWH with normal adiposity (NBF, n = 20) and high adiposity (HBF, n = 26). Spearman's rank-order correlation coefficients were calculated for each pair of variables while adjusting for age, sex, race and legacy d-drugs. Heatmaps display partial Spearman correlation coefficients (rho) only for associations reaching statistical significance ($p < 0.05$).

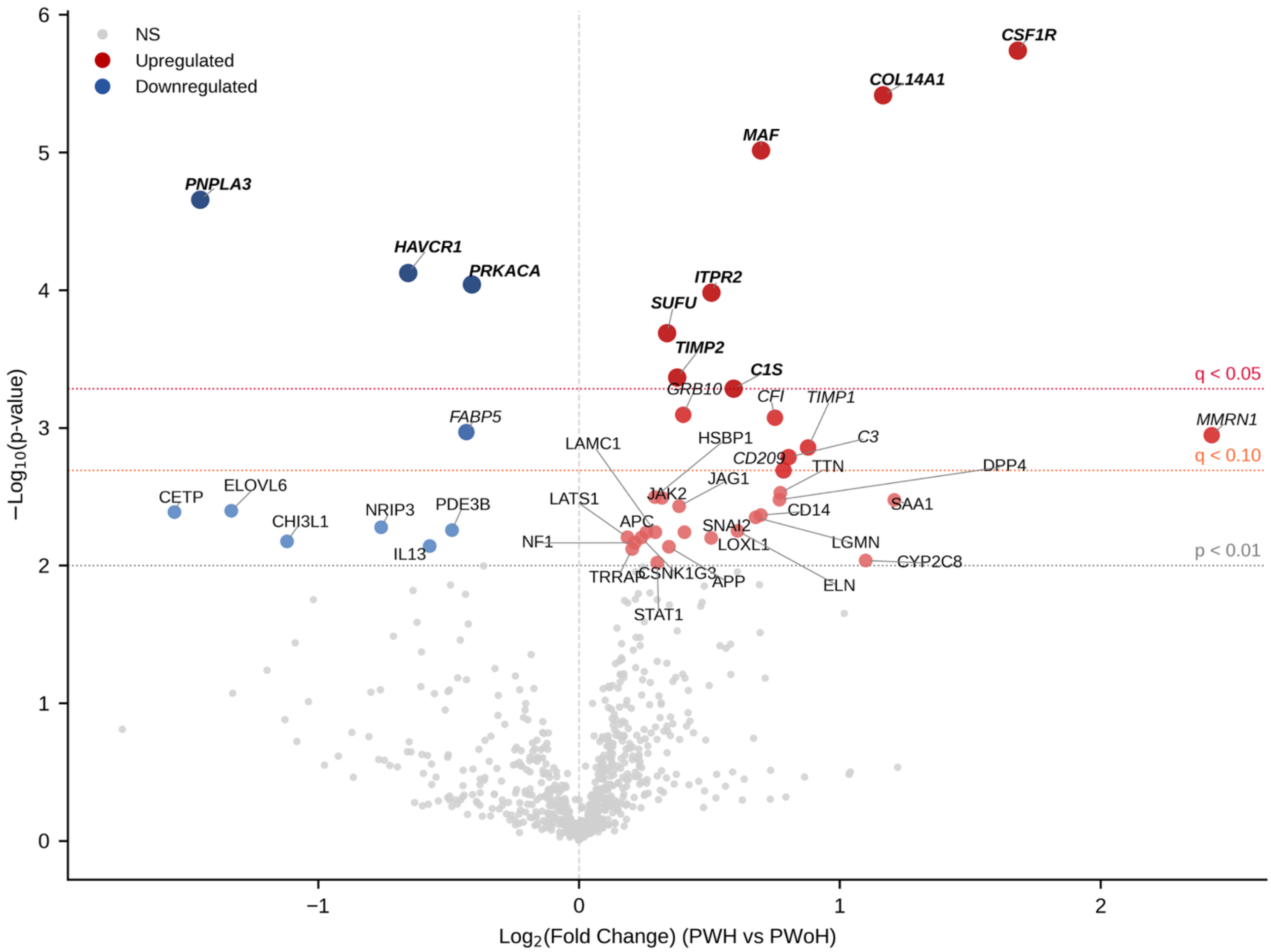

Supplemental Figure 3. Volcano plot of differential gene expression in SAT between PWH and PWoH, with FDR-adjusted thresholds. Each point represents a single gene. Red points indicate upregulated genes and blue points indicate downregulated genes in PWH relative to PWoH; gray points denote genes that did not reach nominal significance. Horizontal dotted lines indicate thresholds for nominal $p < 0.01$ (gray), $q < 0.10$ (orange), and $q < 0.05$ (red). Differential expression was assessed using a negative binomial model adjusting for age, sex, race, batch, and whole-body percent fat. FDR correction was performed using the Benjamini-Hochberg method.

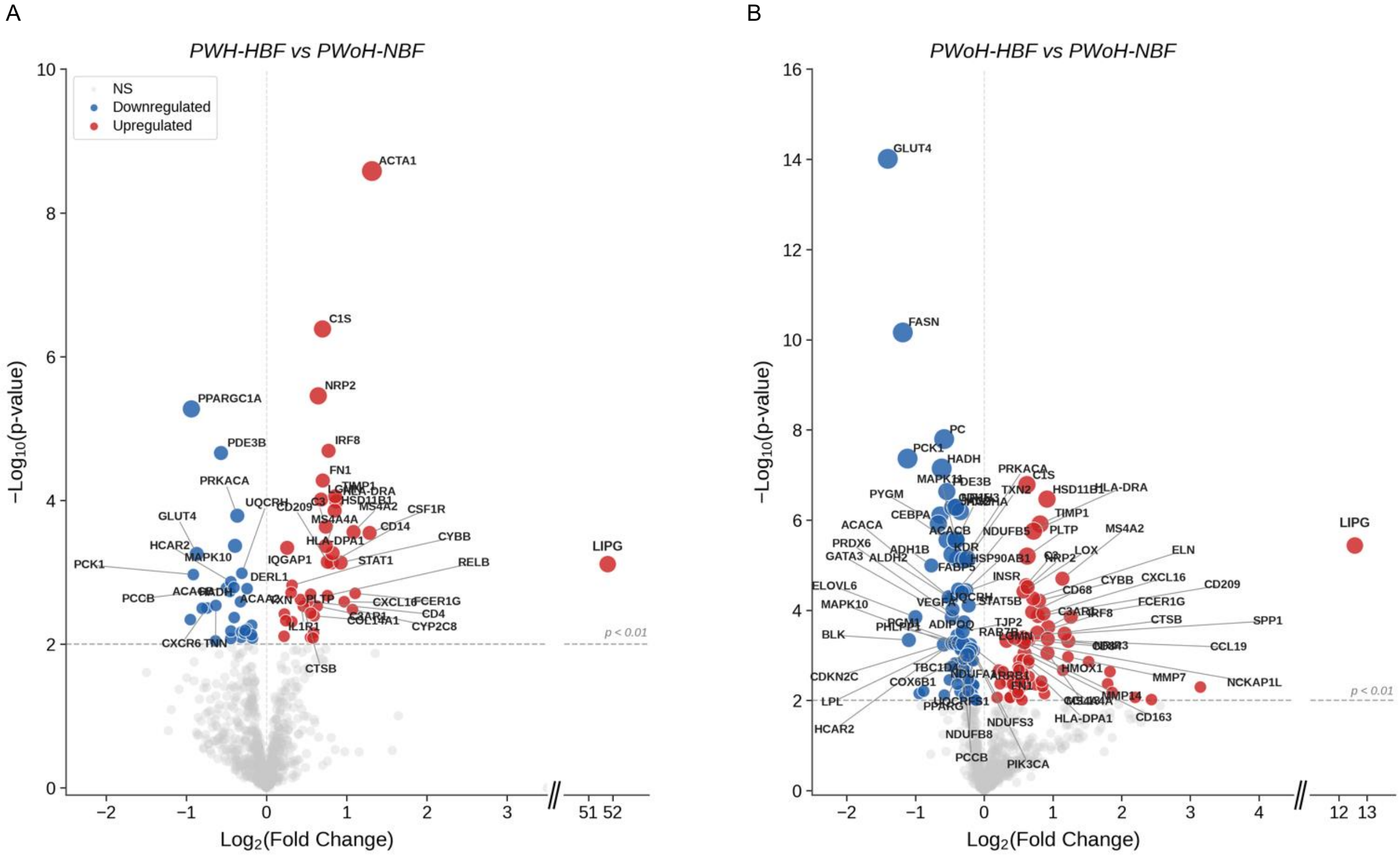

Supplemental Figure 4. Differential gene expression in adipose tissue by HIV and body fat status. Volcano plots showing differential gene expression in subcutaneous adipose tissue. The x-axis shows $\log_2$ fold change, and the y-axis shows $-\log_{10}$ p value. Genes meeting the nominal significance threshold ($p < 0.01$) are highlighted (red, upregulated *relative to PWoH-NBF*; blue, downregulated *relative to PWoH-NBF*) . Dashed line, $p < 0.01$ threshold. A broken x-axis (//) accommodates the LIPG gene. **(A)** PWH-HBF vs PWoH-NBF**. (B)** PWoH-HBF vs PWoH-NBF. Differential expression was analyzed using NanoStringDiff within a covariate-adjusted negative binomial generalized linear modeling framework. Models adjusted for age, sex, race, %BF, and batch.

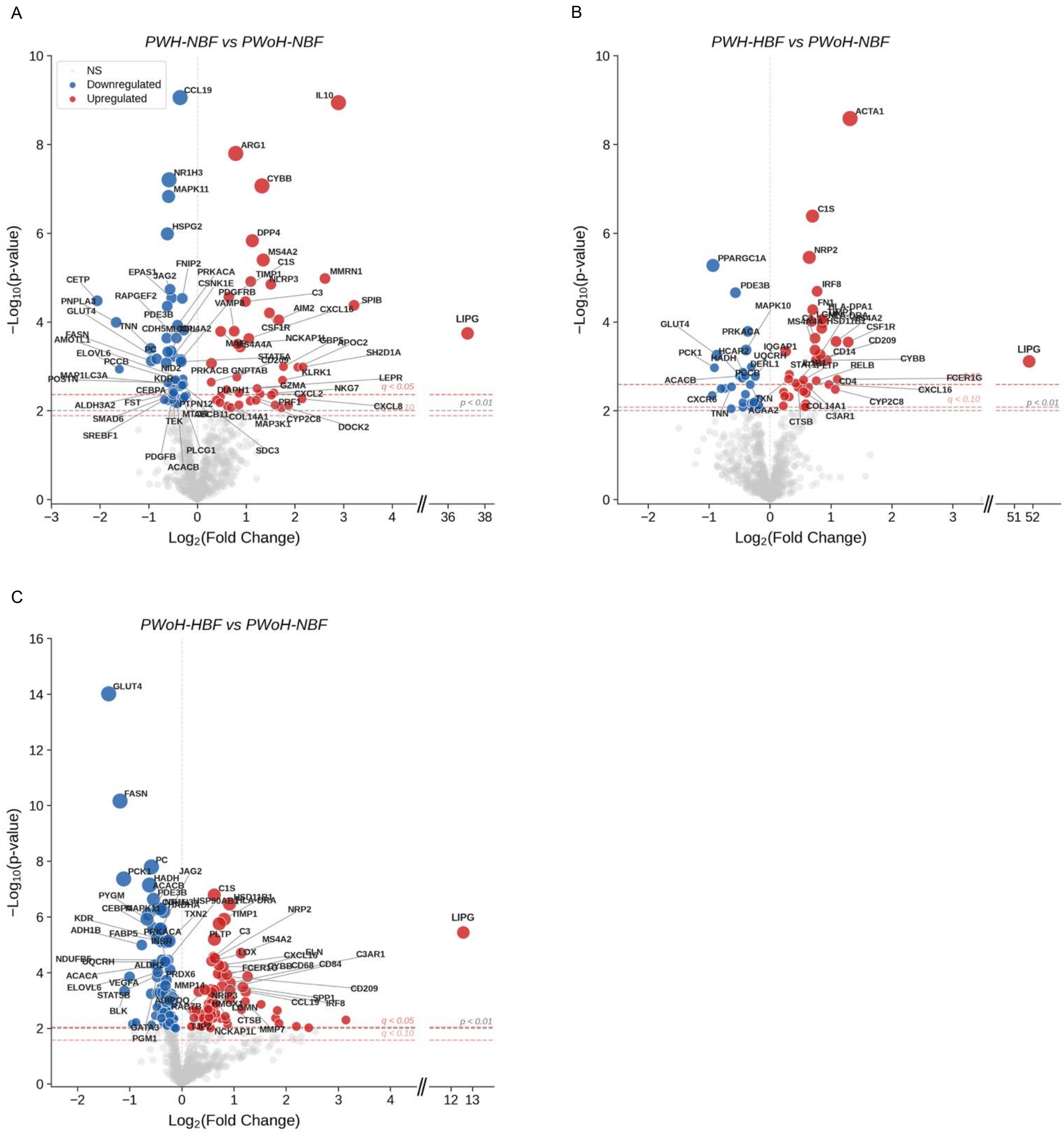

Supplemental Figure 5. Differential gene expression in adipose tissue by HIV and body fat status. Volcano plots showing differential gene expression in subcutaneous adipose tissue. Red, upregulated ($p < 0.01$); blue, downregulated ($p < 0.01$); gray, NS: non-significant. Dashed gray line, $p < 0.01$ threshold; dashed red and orange lines, $q < 0.05$ and $q < 0.10$ thresholds (Benjamini–Hochberg FDR-adjusted p-values). A broken x-axis (//) accommodates the LIPG outlier. **(A)** PWH-NBF vs PWoH-NBF. **(B)** PWH-HBF vs PWoH-NBF. **(C)** PWoH-HBF vs PWoH-NBF. Differential expression was analyzed using NanoStringDiff within a covariate-adjusted negative binomial generalized linear modeling framework. Models adjusted for age, sex, race, batch, and %BF.

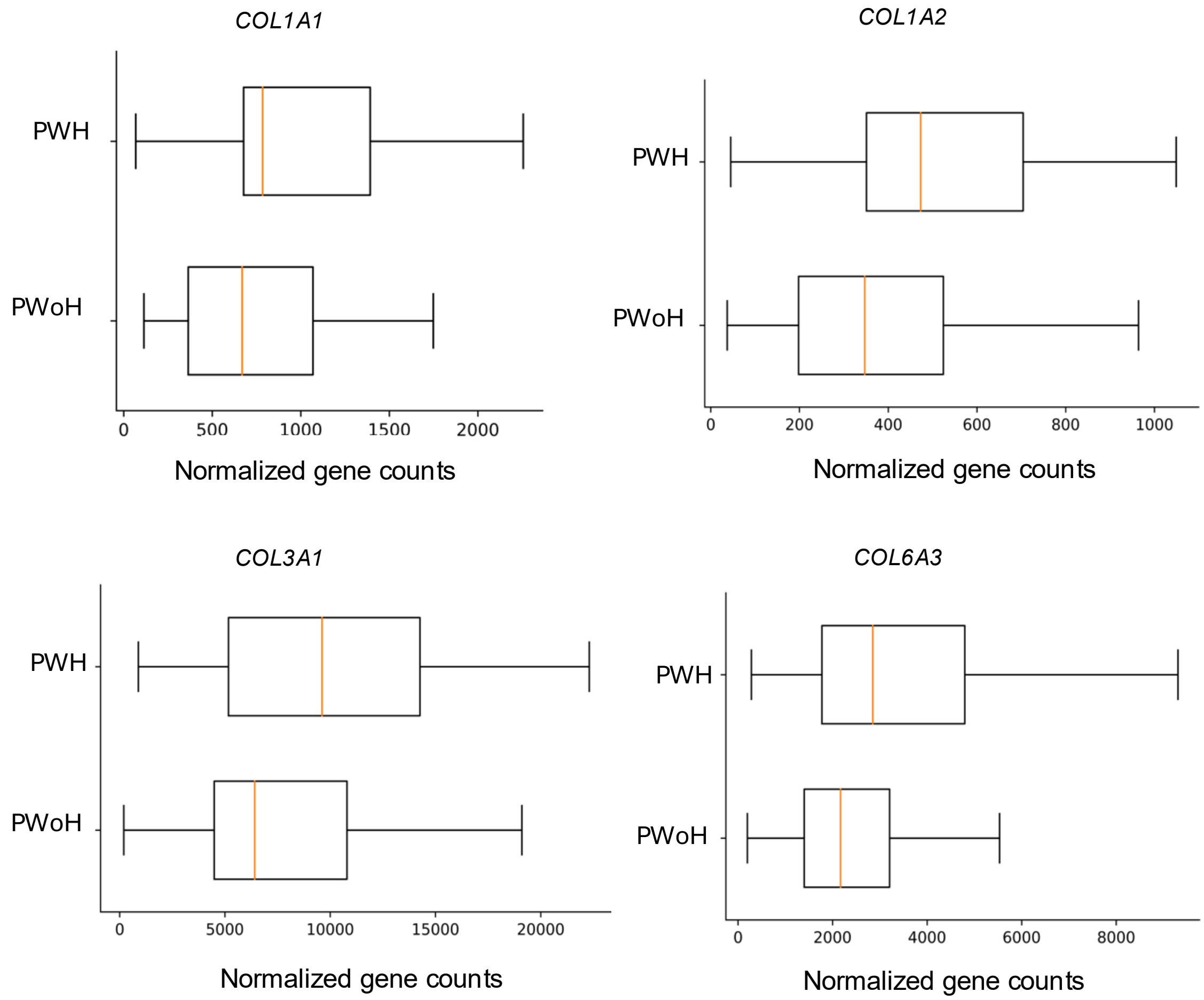

Supplemental Figure 6. Comparison of normalized expression of collagen-encoding transcripts in SAT from PWH and PWoH. NanoString-based quantification of *COL1A1*, *COL1A2*, *COL3A1*, and *COL6A3* expression in SAT samples from PWH and PWoH. Although differences between groups did not reach statistical significance ($p = 0.16$ for *COL1A1*; $p = 0.05$ for *COL1A2*; $p = 0.13$ for *COL3A1*; $p = 0.07$ for *COL6A3*), all transcripts showed a trend toward higher expression in PWH. All boxplot boxes display the interquartile range (IQR), with horizontal lines indicating the median. Statistical comparisons were performed using the Wilcoxon rank-sum test.

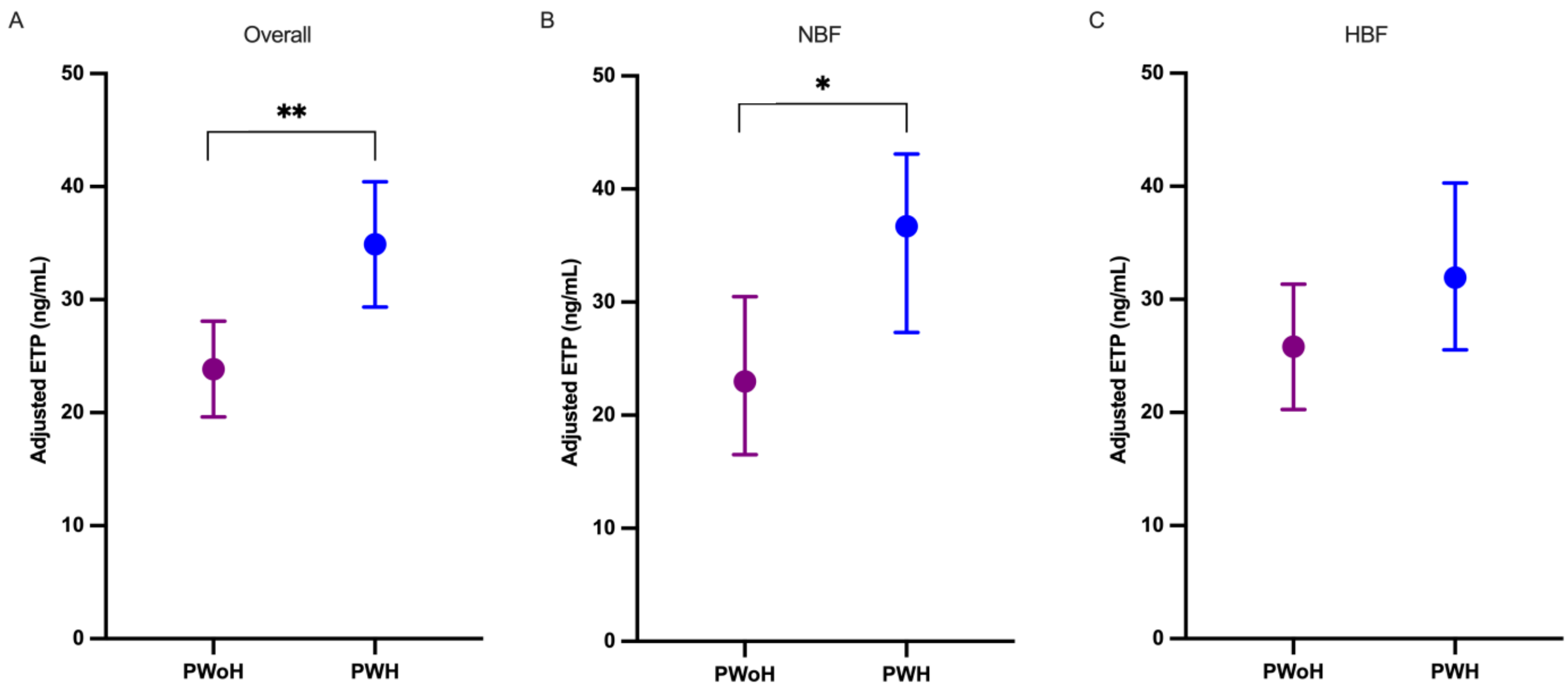

Supplemental Figure 7. Model-derived adjusted estimates of SAT endotrophin (ETP) levels by HIV status. ETP levels were estimated using multivariable linear regression models with HIV status as the primary exposure, adjusting for sex, age, race and %BF, and analyses were stratified by adiposity category as defined in the Methods. **(A)** In the full cohort, PWH exhibited higher adjusted SAT ETP levels compared with PWoH (PWoH n = 73, PWH n = 46; $p < 0.004$). **(B)** Among participants with normal adiposity (NBF) adjusted SAT ETP levels were significantly higher in PWH compared with PWoH (PWoH n = 31, PWH n = 20; $p = 0.041$). **(C)** Among participants with high adiposity (HBF), adjusted SAT ETP levels did not differ between PWH and PWoH (PWoH n = 42, PWH n = 26; $p = 0.16$. Adjusted values are derived from the fitted regression models; error bars represent 95% confidence intervals. P-values correspond to the regression coefficient for HIV status within each model. *$p < 0.05$; ***$p < 0.001$.

Supplemental Tables

Supplemental Table 1. Multivariable linear regression examining associations between HIV status and VAT mass as a proportion of total body mass (%VAT), adjusting for sex, race/ethnicity, and age.

Predictor	β	SE	95% CI	P-value
**HIV Status (PWH vs PWoH)**	0.05	0.06	−0.06, 0.16	0.380
**Sex (Male vs Female)**	0.02	0.05	−0.08, 0.12	0.696
**Age (years)**	0.01	0.00	0.01, 0.02	*<0.001*
**Race: Black**	−0.07	0.07	−0.22, 0.07	0.319
**Race: Other**	−0.04	0.05	−0.014, 0.06	0.445

Although %VAT differed modestly between people with HIV (PWH) and HIV-negative participants in unadjusted analyses (two-sample t test $p = 0.06$), HIV status was not independently associated with %VAT after adjustment for age, sex, and race/ethnicity. Regression coefficients (β) represent the estimated change in the dependent variable per unit increase in each continuous covariate or relative to the reference category for categorical variables. Reference categories: HIV-negative (HIV status), female (sex), White (race). The "Other" race category includes Asian, Native Hawaiian/Pacific Islander, American Indian, and individuals reporting more than one race. Statistically significant associations ($p < 0.05$) are shown in italics. N = 115.

Supplemental Table 2. Multivariable OLS regression examining the relationship between Hydroxyproline levels (HYP) in subcutaneous adipose tissue (SAT) and HIV status, sex, race, age, and whole-body percent fat (N = 120)

**Predictor**	**β**	**95% CI**	**p-value**
**HIV status (PWH vs PWoH)**	*5.05*	*(2.25, 7.84)*	*0.001*
**Sex (Male vs Female)**	*7.46*	*(4.35, 10.57)*	*<0.001*
**Race: Black (vs White)**	0.28	(−3.34, 3.90)	0.878
**Race: Other (vs White)**	1.91	(−0.65, 4.46)	0.142
**Age (years)**	0.02	(−0.08, 0.12)	0.705
**Body fat percentage (%)**	*0.65*	*(0.47, 0.83)*	*<0.001*

HYP was Box-Cox transformed ($\lambda = 0.35$) to satisfy normality and homoscedasticity assumptions prior to model fitting. β coefficients represent the estimated change in transformed hydroxyproline per unit increase in each continuous predictor or relative to the reference category for categorical variables. Reference categories: HIV-negative (PWoH) for HIV status, female for sex, and White for race. “Other” race includes Asian, Native Hawaiian/Pacific Islander, and more than one race. N = 120. Statistically significant associations ($p < 0.05$) are italicized.

Supplemental Table 3. Multivariable OLS regression model restricted to people with HIV (PWH) examining associations between subcutaneous adipose tissue (SAT) hydroxyproline levels antiretroviral treatment characteristics and adiposity (n=46).

**Predictor**	**β**	**95% CI**	**p-value**
Legacy d-drug exposure	6.01	−11.68, 23.70	0.496
INSTI use	−10.77	−33.49, 11.95	0.344
CD4 T-cell count	0.016	−0.017, 0.048	0.344
%Body fat	0.87	−0.29, 2.03	0.137

β coefficients represent the estimated change in transformed hydroxyproline per unit increase in each continuous predictor or relative to the reference category for categorical variables. Reference categories: no exposure for legacy d-drug use, no current use for INSTI, continuous for CD4+ T-cell count and %BF. Hydroxyproline was Box-Cox transformed ($\lambda = 0.58$) to satisfy normality and homoscedasticity assumptions prior to model fitting. Statistically significant associations ($p < 0.05$) are italicized.

Supplemental Table 4. Sequential Sensitivity analyses for the association of ART exposure with subcutaneous adipose tissue hydroxyproline in people with HIV (n=46).

Predictor	β	95% CI	p-value
**Model 1 (Base + Sex)**			
INSTI use	−10.31	−33.34, 12.71	0.371
Legacy d-drug exposure	7.25	−11.32, 25.82	0.435
%Body fat	1.04	−0.32, 2.40	0.130
CD4 T-cell count	0.018	−0.017, 0.052	0.305
Sex (Male)	5.75	−17.80, 29.30	0.624
**Model 2 (Base + Age)**			
INSTI use	−11.33	−34.39, 11.73	0.327
Legacy d-drug exposure	6.97	−11.31, 25.25	0.445
%Body fat	0.88	−0.30, 2.05	0.139
CD4 T-cell count	0.015	−0.018, 0.049	0.352
Age	−0.19	−0.96, 0.58	0.618
**Model 3 (Base + Race)**			
INSTI use	−8.96	−31.86, 13.93	0.433
Legacy d-drug exposure	5.16	−12.72, 23.04	0.563
%Body fat	0.87	−0.29, 2.04	0.137
CD4 T-cell count	0.019	−0.015, 0.053	0.274
Race: Black	6.65	−11.04, 24.33	0.452
Race: Other	14.19	−5.63, 34.00	0.156

Each model extends the base model (legacy d-drug exposure, INSTI use, CD4+ T-cell count, %BF) with an additional covariate. Hydroxyproline was Box-Cox transformed ($\lambda = 0.58$) prior to model fitting. β coefficients represent the estimated change in transformed hydroxyproline per unit increase in each continuous predictor or relative to the reference category for categorical variables. Reference categories: no exposure for legacy d-drug use, no current use for INSTI, female for sex, White for race. “Other” race includes Asian, Native Hawaiian/Pacific Islander, and more than one race. Statistically significant associations ($p < 0.05$) are italicized

Supplemental Table 5. Demographic and clinical characteristics of the study population stratified by adiposity and HIV status.

	Normal Adiposity, NBF		High Adiposity, HBF	
**Parameter**	**PWoH (n=31)**	**PWH (n=20)**	**PWoH (n=43)**	**PWH (n=26)**
**Age (years)**	44 ± 14	52 ± 10	50 ± 12	52 ± 11
**Sex at birth (Female/Male)**	17/14	3/17	22/21	7/19
**Race**				
Asian	14	1	15	0
Black	2	6	1	10
White	13	7	20	12
More than one race	1	4	6	1
Other	1	2	1	3
**Ethnicity**				
Hispanic or Latino	2	4	10	6
Not Hispanic or Latino	29	16	32	20
Unknow	0	0	1	0
**BMI (kg/m^2)**	23.4 ± 3.1	25.6 ± 3.4	32.7 ± 8.2	32.9 ± 10.1
**Body Fat Percentage (%)**	25.7 ± 6.0	23.2 ± 3.1	36.0 ± 7.0	34.2 ± 7.0
**Fasting glucose (mg/dL)**	92 (85, 100)	98 (88, 107)	93 (88,101)	88 (84, 98)
**Fasting insulin (mU/L)** [A]	5.8 (3.9, 9.5)	9.1 (7.2, 14.4)	14.6 (9.2, 24.4)	11.6 (7.0, 16.7)
**HOMA-IR**[A]	1.4 (0.8, 2.8)	2.4 (1.7, 3.6)	4.0 (2.0, 6.3)	2.7 (1.5, 4.0)
**HbA1c (%)**	5.4 ± 0.5	5.6 ± 0.4	5.8 ± 0.7	5.3 ± 0.5
**Duration of HIV (years)**	-	23.8 ± 9.8	-	20.5 ± 12.9
**Duration of ART use (years)**	-	11.8 ± 9.0	-	8.6 ± 9.3
**CD4$^+$ cell count (cells/mm^3)**	-	537.0 (471.8, 774.2)	-	713.5 (605.2, 815.0)
**ART regimen**				
**INSTI, n (%)**	-	18 (90%)	-	22 (85%)
**NNRTI, n (%)**	-	5 (25%)	-	5 (19%)
**Protease Inhibitor, n (%)**	-	3 (15%)	-	1 (4%)
**Exposure to d-drugs, n (%)**	-	10 (50%)	-	4 (15%)
**Hydroxyproline (ng/mg fat)**	72.7 (48.7, 166.5)	448.4 (287.3, 815.9)	389.7 (271.9, 842.5)	654.0 (405.0, 863.1)

Males were classified as NBF (%BF <25%) or HBF (%BF ≥25%); females were classified as NBF (%BF <35%) or HBF (%BF ≥35%). Values are presented as mean ± SD or median (interquartile range). Other category for race includes one individual identifying as American Indian, one as Native Hawaiian or Pacific Islander, and five with unknown or not reported race. Missing data: n = 5 (VAT/total mass), n = 2 (fasting insulin and HOMA-IR). PWH, people with HIV; PWoH, people without HIV; NBF, normal body fat; HBF, high body fat; BMI, body mass index; VAT, visceral adipose tissue; HOMA-IR, homeostatic model assessment for insulin resistance; HbA1c, hemoglobin A1c; INSTI, integrase strand transfer inhibitor; NNRTI, non-nucleoside reverse transcriptase inhibitor.

Supplemental Table 6. Characteristics of Study Participants included in SAT NanoString transcriptional analysis.

Parameter	PWoH (n=38)	PWH (n=37)	p-value
**Age (years)**	48.5 ± 13.2	52.2 ± 10.8	0.23
**Sex at birth**			0.07
**Female**	20	10	
**Male**	18	27	
**Race**			*<0.01*
Asian	15	1	
Black	2	15	
White	15	16	
More than one race	6	3	
Other*	0	2	
**Ethnicity**			0.75
Hispanic or Latino	6	6	
Not Hispanic or Latino	31	31	
Unknown	1	0	
**BMI (kg/m²)**	29.1 ± 8.0	29.7 ± 7.7	0.47
**Body Fat Percentage (%)**	32.1 ± 8.1	29.9 ± 7.8	0.18
**VAT mass/total mass (%)^{A}**	0.7 ± 0.3	0.7 ± 0.3	0.48
**Fasting glucose (mg/dL)**	93.0 (87.5, 98.5)	90.5 (84.8, 99.5)	0.39
**Fasting insulin (mU/L)^{A}**	11.8 (6.2, 19.3)	9.9 (6.8, 16.6)	0.60
**HOMA-IR^{A}**	2.9 (1.4, 4.4)	2.6 (1.4, 3.7)	0.43
**Duration of HIV (years)**	—	23.1 (15.0, 31.2)	
**Duration of ART use (years)**	—	5.6 (2.4, 13.1)	
**$CD4^{+}$ cell count (cells/mm^{3})**	—	631.0 (521.0, 830.2)	
**ART regimen**			
**INSTI, n (%)**	—	32 (89%)	
**NNRTI, n (%)**	—	6 (17%)	
**PI, n (%)**	—	3 (8%)	
**Exposure to d-drugs, n (%)**	-	9 (25%)	-
**Hydroxyproline (ng/mg fat)**	290.7 (79.6, 757.2)	507.2 (335.8, 877.8)	*0.02*

Values are presented as mean ± SD or median (interquartile range). Differences between the groups were analyzed by Chi-Square test for categorical variables and Wilcoxon rank-sum test for continuous variables. *Other category for race includes one individual identifying as Native Hawaiian or Pacific Islander and one with unknown or not reported race. ^{A}Missing data: n = 4 (VAT/total mass), n = 2 (fasting insulin, HOMA-IR). PWH, people with HIV; PWoH, people without HIV; BMI, body mass index; VAT, visceral AT; HOMA-IR, homeostatic model assessment for insulin resistance: HbA1c, hemoglobin A1c; ART, antiretroviral therapy; INSTI, integrase strand transfer inhibitor; NNRTI, non-nucleoside reverse transcriptase inhibitor. ART regimen categories are not mutually exclusive.

Supplemental Table 7. Characteristics of Study Participants included in SAT NanoString transcriptional analysis, stratified by adiposity and HIV status.

	Normal Adiposity, NBF		High Adiposity, HBF	
**Parameter**	**PWoH (n=18)**	**PWH (n=16)**	**PWoH (n=20)**	**PWH (n=21)**
**Age (years)**	45.6 ± 13.2	52.3 ± 10.6	50.5 ± 13.1	52.1 ± 11.1
**Sex at birth (Female/Male)**	9/9	3/13	11/9	7/14
**Race**				
Asian	7	1	8	0
Black	1	5	1	10
White	7	6	8	10
More than one race	3	3	3	0
Other*	0	1	0	1
**Ethnicity**				
Hispanic or Latino	2	3	4	3
Not Hispanic or Latino	16	13	15	18
Unknown	0	0	1	0
**BMI (kg/m²)**	24.1 ± 3.2	25.1 ± 2.4	32.5 ± 8.5	32.9 ± 8.6
**Body Fat Percentage (%)**	26.1 ± 5.6	23.4 ± 3.3	36.2 ± 6.9	34.5 ± 6.8
**Fasting glucose (mg/dL)**	95.5 (87.5, 104.5)	98.0 (87.5, 107.0)	92.0 (87.5, 95.0)	88.0 (84.0, 95.0)
**Fasting insulin (mU/L)** [A]	5.7 (3.7, 13.0)	8.9 (6.8, 11.8)	17.1 (9.3, 25.2)	12.3 (7.2, 17.4)
**HOMA-IR** [A]	1.4 (0.8, 3.2)	2.2 (1.5, 3.2)	4.0 (2.1, 5.9)	2.8 (1.5, 3.8)
**HbA1c (%)**	5.6 ± 0.4	5.5 ± 0.4	5.6 ± 0.4	5.3 ± 0.5
**Duration of HIV (years)**	—	23.6 ± 9.7	—	21.6 ± 13.6
**Duration of ART use (years)**	—	11.6 ± 9.3	—	8.5 ± 9.9
**$CD4^+$ cell count (cells/mm^3)**	—	536.0 (450.5, 680.0)	—	774.0 (624.0, 864.0)
**ART regimen**				
**INSTI, n (%)**	—	15 (100%)	—	17 (81%)
**NNRTI, n (%)**	—	1 (7%)	—	5 (24%)
**Protease Inhibitor, n (%)**	—	2 (13%)	—	1 (5%)
**Exposure to d-drugs, n (%)**	—	7 (47%)	—	2 (10%)
**Hydroxyproline (ng/mg fat)**	100.6 (55.3, 205.6)	410.4 (268.9, 687.5)	375.6 (271.9, 915.0)	657.8 (423.9, 899.5)

Adiposity classification: males were classified as NBF (%BF <25%) or HBF (%BF ≥25%); females were classified as NBF (%BF <35%) or HBF (%BF ≥35%). Values are presented as mean ± SD or median (interquartile range). *Other category for race includes one individual identifying as Native Hawaiian or Pacific Islander and one with unknown or not reported race. [A] Missing data: n = 4 (VAT/total mass), n = 2 (fasting insulin, HOMA-IR). PWH, people with HIV; PWoH, people without HIV; NBF, normal body fat; HBF, high body fat; BMI, body mass index; VAT, visceral adipose tissue; HOMA-IR, homeostatic model assessment for insulin resistance; HbA1c, hemoglobin A1c; INSTI, integrase strand transfer inhibitor; NNRTI, non-nucleoside reverse transcriptase inhibitor.

Supplemental Table 8. Genes differentially expressed in adipose tissue across groups compared to the reference group (PWoH-NBF).

A. DGE analysis of PWH-NBF group vs control (PWoH-NBF). Total significant genes ($p < 0.01$): 89; Upregulated: 44; Downregulated: 45

Upregulated genes (n = 44)

Gene	logFC	p-value	q-value
**IL10**	2.8961	1.15e-9	4.36e-7
**ARG1**	0.7873	1.60e-8	4.05e-6
**CYBB**	1.3255	8.60e-8	1.31e-5
**DPP4**	1.1278	1.48e-6	1.40e-4
**MS4A2**	1.3512	4.02e-6	3.40e-4
**MMRN1**	2.6200	1.05e-5	7.99e-4
**TIMP1**	1.0954	1.23e-5	8.49e-4
**NLRP3**	1.5083	1.42e-5	8.97e-4
**C1S**	0.6465	2.74e-5	0.0014
**C3**	0.9872	3.48e-5	0.0015
**SPIB**	3.2162	4.23e-5	0.0017
**CSF1R**	1.4787	6.27e-5	0.0023
**AIM2**	1.6723	8.99e-5	0.0031
**VAMP8**	0.7558	1.62e-4	0.0046
**MAF**	0.4776	1.63e-4	0.0046
**LIPG**	37.0635	1.79e-4	0.0049
**CXCL16**	1.0558	2.36e-4	0.0058
**NCKAP1L**	0.8280	3.15e-4	0.0075
**MS4A4A**	0.8807	3.67e-4	0.0084
**PRKACB**	0.2885	8.57e-4	0.0151
**GBP5**	1.7662	0.0010	0.0173
**SH2D1A**	2.1685	0.0010	0.0173
**APOC2**	2.0627	0.0010	0.0173
**CD209**	0.8098	0.0017	0.0275
**KLRK1**	1.7486	0.0021	0.0301
**GNPTAB**	0.2843	0.0023	0.0326
**GZMA**	1.2260	0.0032	0.0406
**LEPR**	0.5996	0.0034	0.0426
**NKG7**	1.5701	0.0040	0.0476
**DOCK2**	0.8559	0.0040	0.0476
**CXCL2**	1.2861	0.0042	0.0491
**PRF1**	1.5200	0.0044	0.0503
**DIAPH1**	0.4731	0.0049	0.0528
**CXCL8**	2.1480	0.0054	0.0557
**MAP3K1**	0.3894	0.0058	0.0567
**CYP2C8**	1.2042	0.0058	0.0567
**CXCR4**	1.0848	0.0061	0.0576
**PELI1**	0.4620	0.0066	0.0618
**MS4A1**	0.8535	0.0074	0.0665
**EGR1**	1.5963	0.0074	0.0665
**PTGS2**	1.8701	0.0076	0.0670
**COL14A1**	0.6150	0.0079	0.0688
**IRF8**	0.6902	0.0085	0.0734
**CCL4**	1.7254	0.0086	0.0734

Downregulated genes (n = 45)

Gene	logFC	p-value	q-value
**CCL19**	-0.3549	8.80e-10	4.36e-7
**NR1H3**	-0.5822	6.27e-8	1.19e-5
**MAPK11**	-0.5919	1.49e-7	1.89e-5
**HSPG2**	-0.6163	1.04e-6	1.13e-4
**JAG2**	-0.5612	1.84e-5	0.0011
**EPAS1**	-0.5292	2.89e-5	0.0014
**FNIP2**	-0.3101	2.95e-5	0.0014
**CETP**	-2.0569	3.33e-5	0.0015
**PDE3B**	-0.6200	4.43e-5	0.0017
**PNPLA3**	-1.6681	1.02e-4	0.0034
**PRKACA**	-0.4078	1.18e-4	0.0037
**CSNK1E**	-0.2798	1.53e-4	0.0046
**RAPGEF2**	-0.4307	2.30e-4	0.0058
**CDH5**	-0.6293	2.32e-4	0.0058
**TNN**	-0.9571	3.93e-4	0.0088
**MLXIPL**	-0.5405	4.51e-4	0.0098
**PC**	-0.6087	4.72e-4	0.0100
**COL4A2**	-0.4985	5.98e-4	0.0123
**GLUT4**	-0.8365	6.79e-4	0.0136
**PDGFRB**	-0.3369	7.06e-4	0.0138
**FASN**	-0.9488	7.61e-4	0.0145
**STAT5A**	-0.3332	7.85e-4	0.0145
**NID2**	-0.6404	8.37e-4	0.0151
**ELOVL6**	-1.6034	0.0012	0.0188
**AMOTL1**	-0.2947	0.0019	0.0299
**PCCB**	-0.4434	0.0020	0.0301
**KDR**	-0.4584	0.0020	0.0301
**POSTN**	-0.6099	0.0023	0.0329
**MAP1LC3A**	-0.2830	0.0026	0.0355
**CEBPA**	-0.5357	0.0026	0.0355
**SDC3**	-0.3137	0.0027	0.0357
**ALDH3A2**	-0.3526	0.0029	0.0378
**TEK**	-0.4872	0.0039	0.0476
**FST**	-0.5163	0.0039	0.0476
**SMAD6**	-0.5027	0.0043	0.0500
**ABCB11**	-0.2295	0.0046	0.0511
**PTPN12**	-0.2613	0.0048	0.0528
**MTOR**	-0.2671	0.0052	0.0552
**ACACB**	-0.4198	0.0052	0.0552
**PLCG1**	-0.2916	0.0054	0.0557
**PDGFB**	-0.5216	0.0056	0.0566
**SREBF1**	-0.6769	0.0057	0.0566
**COX4I2**	-0.6023	0.0059	0.0572
**MAP2K1**	-0.4588	0.0068	0.0629
**PDE2A**	-0.4154	0.0070	0.0644

B. DGE analysis of PWH-HBF group vs control (PWoH-NBF). Total significant genes ($p < 0.01$): 68; Upregulated: 39; Downregulated: 29

Upregulated genes (n = 39)

Gene	logFC	p-value	q-value
**ACTA1**	1.3152	2.62e-9	1.99e-6
**C1S**	0.6989	4.12e-7	1.57e-4
**NRP2**	0.6456	3.51e-6	8.90e-4
**IRF8**	0.7741	2.04e-5	0.0028
**FN1**	0.7019	5.29e-5	0.0057
**TIMP1**	0.8571	8.75e-5	0.0079
**LGMN**	0.6810	9.74e-5	0.0079
**HLA-DRA**	0.8718	1.03e-4	0.0079
**HSD11B1**	0.8502	1.39e-4	0.0096
**C3**	0.7400	2.32e-4	0.0136
**MS4A2**	1.0858	2.76e-4	0.0144
**CSF1R**	1.2861	2.84e-4	0.0144
**MS4A4A**	0.7397	4.31e-4	0.0193
**IQGAP1**	0.2581	4.57e-4	0.0193
**HLA-DPA1**	0.8248	5.43e-4	0.0211
**CD209**	0.7645	7.16e-4	0.0242
**CD14**	0.8125	7.28e-4	0.0242
**CYBB**	0.9252	7.33e-4	0.0242
**LIPG**	51.7935	7.67e-4	0.0243
**STAT1**	0.3191	0.0015	0.0412
**TXN**	0.3071	0.0019	0.0439
**FCER1G**	1.1062	0.0020	0.0439
**PLTP**	0.5514	0.0020	0.0440
**RELB**	0.7604	0.0021	0.0446
**IL1R1**	0.4234	0.0024	0.0489
**CD4**	0.5132	0.0025	0.0489
**CXCL16**	0.9695	0.0026	0.0489
**C3AR1**	0.6332	0.0030	0.0524
**CTSB**	0.4590	0.0030	0.0524
**CYP2C8**	1.0730	0.0033	0.0547
**VAMP8**	0.5530	0.0037	0.0600
**CTNNB1**	0.2236	0.0038	0.0604
**COL14A1**	0.5948	0.0040	0.0627
**LAMC1**	0.2411	0.0047	0.0689
**GNB4**	0.3153	0.0049	0.0699
**LIPA**	0.5874	0.0070	0.0893
**PDGFRB**	0.2193	0.0078	0.0947
**CD163**	0.5491	0.0081	0.0947
**NCKAP1L**	0.5794	0.0082	0.0947

Downregulated genes (n = 29)

Gene	logFC	p-value	q-value
**PPARGC1A**	-0.9364	5.34e-6	0.0010
**PDE3B**	-0.5672	2.19e-5	0.0028
**PRKACA**	-0.3641	1.63e-4	0.0103
**MAPK10**	-0.3920	4.29e-4	0.0193
**GLUT4**	-0.8697	5.56e-4	0.0211
**UQCRH**	-0.3071	0.0010	0.0314
**PCK1**	-0.9120	0.0011	0.0315
**HCAR2**	-0.4439	0.0014	0.0382

**PCCB**	-0.4057	0.0016	0.0415
**ACACB**	-0.4999	0.0017	0.0415
**DERL1**	-0.2416	0.0017	0.0415
**HADH**	-0.4562	0.0019	0.0439
**ACAA2**	-0.3243	0.0026	0.0489
**TNN**	-0.6311	0.0029	0.0524
**CXCR6**	-0.7411	0.0031	0.0536
**FASN**	-0.8017	0.0032	0.0536
**PGM1**	-0.4025	0.0043	0.0649
**PNPLA3**	-0.9494	0.0046	0.0679
**CHUK**	-0.1849	0.0055	0.0769
**HIKESHI**	-0.2500	0.0062	0.0855
**UQCRFS1**	-0.2659	0.0065	0.0876
**PC**	-0.4433	0.0066	0.0876
**PHLPP1**	-0.3096	0.0068	0.0887
**SERPINF1**	-0.2739	0.0070	0.0893
**TXN2**	-0.1856	0.0074	0.0925
**FABP5**	-0.3158	0.0081	0.0947
**PPM1A**	-0.1751	0.0083	0.0947
**FST**	-0.4450	0.0084	0.0947
**IL13**	-0.6363	0.0090	0.1010

C. DGE analysis of PWoH-HBF group vs control (PWoH-NBF). Total significant genes ($p < 0.01$): 148; Upregulated: 70; Downregulated: 78

Upregulated genes (n = 70)

Gene	logFC	p-value	q-value
**C1S**	0.6236	1.64e-7	2.08e-5
**HSD11B1**	0.9152	3.44e-7	3.26e-5
**HLA-DRA**	0.8139	1.22e-6	6.65e-5
**TIMP1**	0.7189	1.76e-6	8.94e-5
**LIPG**	12.5659	3.62e-6	1.45e-4
**PLTP**	0.6263	6.32e-6	2.29e-4
**MS4A2**	1.1392	2.00e-5	5.85e-4
**C3**	0.6112	2.79e-5	7.85e-4
**NRP2**	0.6326	3.07e-5	8.32e-4
**LOX**	0.5728	3.84e-5	9.34e-4
**ELN**	0.7150	5.49e-5	0.0012
**CYBB**	0.7993	6.14e-5	0.0013
**CD68**	0.6992	1.09e-4	0.0021
**CXCL16**	0.8650	1.19e-4	0.0022
**CD209**	0.7840	1.35e-4	0.0024
**FCER1G**	1.2622	1.40e-4	0.0024
**C3AR1**	0.9329	2.37e-4	0.0038
**IRF8**	0.7731	3.15e-4	0.0049
**SPP1**	1.1718	3.31e-4	0.0050
**CTSB**	0.5693	4.02e-4	0.0059
**RAB7B**	0.4428	4.10e-4	0.0059
**NRIP3**	0.9268	4.33e-4	0.0060
**LGMN**	0.6207	4.35e-4	0.0060
**CD84**	0.6423	4.58e-4	0.0061
**MMP14**	0.3262	4.78e-4	0.0062
**CCL19**	1.2252	4.80e-4	0.0062
**NCKAP1L**	0.5801	5.19e-4	0.0064
**HMOX1**	0.5864	5.20e-4	0.0064

**CCL13**	0.9219	8.95e-4	0.0092
**CD163**	0.5885	9.05e-4	0.0092
**MMP7**	1.2207	0.0011	0.0106
**HLA-DPA1**	0.5608	0.0013	0.0119
**MS4A4A**	0.6493	0.0013	0.0119
**FN1**	0.5062	0.0013	0.0119
**SERPINE1**	1.5204	0.0014	0.0122
**LIPA**	0.6483	0.0015	0.0129
**S100A4**	0.4946	0.0019	0.0158
**VAMP8**	0.5088	0.0021	0.0172
**AP2S1**	0.2137	0.0021	0.0172
**NLRP3**	1.1444	0.0022	0.0173
**ATP6V1G2**	1.8316	0.0023	0.0180
**CTSD**	0.2766	0.0023	0.0180
**LOXL4**	0.5650	0.0025	0.0189
**ADCY7**	0.5109	0.0028	0.0214
**FBP1**	0.6532	0.0030	0.0218
**ANGPTL4**	0.3924	0.0030	0.0219
**NCEH1**	0.5736	0.0034	0.0238
**THBS1**	0.8349	0.0037	0.0258
**ADAM9**	0.3295	0.0039	0.0266
**SYK**	0.5243	0.0040	0.0271
**TXN**	0.2331	0.0043	0.0282
**ACSM3**	1.7979	0.0043	0.0282
**LOXL1**	0.4136	0.0044	0.0282
**CD14**	0.6026	0.0045	0.0284
**CCN2**	0.7503	0.0045	0.0284
**CYP27A1**	0.5031	0.0047	0.0293
**MMP9**	0.8497	0.0049	0.0299
**C9**	3.1466	0.0051	0.0308
**FGD2**	0.4360	0.0051	0.0308
**RELB**	0.5090	0.0060	0.0359
**CCL4**	1.8639	0.0067	0.0383
**SCIN**	0.4900	0.0067	0.0383
**IL12RB2**	0.8784	0.0071	0.0400
**LEP**	0.5090	0.0080	0.0438
**AMOTL2**	0.3766	0.0086	0.0458
**ADH6**	2.1973	0.0086	0.0458
**CD4**	0.3783	0.0086	0.0458
**TIMP2**	0.1896	0.0086	0.0458
**CFHR5**	2.4348	0.0096	0.0503
**PLCB2**	0.5505	0.0098	0.0508

Downregulated genes (n = 78)

Gene	logFC	p-value	q-value
**GLUT4**	-1.4010	9.77e-15	7.43e-12
**FASN**	-1.1828	6.92e-11	2.63e-8
**PC**	-0.5810	1.60e-8	4.07e-6
**PCK1**	-1.1136	4.33e-8	8.24e-6
**HADH**	-0.6168	7.19e-8	1.09e-5
**PDE3B**	-0.5423	2.33e-7	2.53e-5
**CDH5**	-0.4566	5.20e-7	3.97e-5
**NR1H3**	-0.4114	5.22e-7	3.97e-5
**HADHA**	-0.3448	6.49e-7	4.48e-5
**ACACB**	-0.6410	7.77e-7	4.92e-5

**PYGM**	-0.6661	1.21e-6	6.65e-5
**MAPK11**	-0.4086	2.73e-6	1.23e-4
**CEBPA**	-0.5381	2.77e-6	1.23e-4
**JAG2**	-0.4067	2.90e-6	1.23e-4
**KDR**	-0.4664	5.78e-6	2.20e-4
**FABP5**	-0.3853	7.31e-6	2.43e-4
**TXN2**	-0.2448	7.34e-6	2.43e-4
**PRKACA**	-0.3003	7.69e-6	2.43e-4
**ADH1B**	-0.7668	1.02e-5	3.10e-4
**NDUFB5**	-0.3771	3.49e-5	9.12e-4
**HSP90AB1**	-0.2589	3.60e-5	9.12e-4
**INSR**	-0.2935	4.00e-5	9.34e-4
**UQCRH**	-0.3219	4.06e-5	9.34e-4
**ALDH2**	-0.5134	5.14e-5	0.0011
**ACACA**	-0.4791	5.47e-5	0.0012
**STAT5B**	-0.2243	7.87e-5	0.0016
**VEGFA**	-0.4594	9.63e-5	0.0019
**ELOVL6**	-0.9998	1.43e-4	0.0024
**GATA3**	-0.4702	1.43e-4	0.0024
**PRDX6**	-0.2926	1.86e-4	0.0031
**TJP2**	-0.3128	2.95e-4	0.0047
**ADIPOQ**	-0.3874	3.69e-4	0.0055
**BLK**	-1.0948	4.59e-4	0.0061
**PGM1**	-0.3668	5.28e-4	0.0064
**HCAR2**	-0.4585	5.37e-4	0.0064
**LPL**	-0.4208	5.38e-4	0.0064
**PHLPP1**	-0.3058	5.56e-4	0.0065
**CDKN2C**	-0.5854	5.77e-4	0.0066
**ARRB1**	-0.2037	5.90e-4	0.0067
**NDUFA1**	-0.2396	6.70e-4	0.0074
**MAPK10**	-0.3277	6.70e-4	0.0074
**PCCB**	-0.3310	7.23e-4	0.0078
**PIK3CA**	-0.1894	7.47e-4	0.0080
**NDUFS3**	-0.1619	7.85e-4	0.0083
**TBC1D4**	-0.2145	8.54e-4	0.0089
**UQCRFS1**	-0.2440	9.87e-4	0.0099
**COX6B1**	-0.2515	0.0010	0.0103
**PPARG**	-0.2958	0.0012	0.0117
**NDUFB8**	-0.1792	0.0013	0.0119
**SMAD4**	-0.1573	0.0013	0.0120
**AMOTL1**	-0.2485	0.0013	0.0120
**FLT4**	-0.4274	0.0015	0.0129
**FABP4**	-0.3652	0.0015	0.0129
**IRS1**	-0.4752	0.0018	0.0148
**MMUT**	-0.2952	0.0019	0.0158
**CAT**	-0.2750	0.0022	0.0176
**NOTCH4**	-0.3443	0.0027	0.0202
**COX4I2**	-0.3738	0.0029	0.0214
**EPAS1**	-0.2324	0.0032	0.0235
**ANAPC1**	-0.2002	0.0033	0.0237
**PLCG1**	-0.2298	0.0034	0.0240
**C5**	-0.5046	0.0035	0.0245
**COX6A1**	-0.1978	0.0040	0.0271
**SLC25A10**	-0.3887	0.0044	0.0282
**CSNK1E**	-0.1994	0.0044	0.0282
**DEPDC5**	-0.1524	0.0046	0.0286

**CUL1**	-0.1475	0.0048	0.0298
**COX7C**	-0.2631	0.0059	0.0351
**STAT5A**	-0.2292	0.0062	0.0364
**SCD**	-0.8793	0.0062	0.0365
**MLXIPL**	-0.3500	0.0063	0.0367
**CETP**	-0.9444	0.0070	0.0399
**GSK3B**	-0.1709	0.0074	0.0412
**DLL1**	-0.5786	0.0076	0.0423
**PDHA1**	-0.2799	0.0080	0.0439
**SMARCC2**	-0.1469	0.0093	0.0492
**NDUFC1**	-0.1354	0.0098	0.0509
**SUGT1**	-0.1185	0.0099	0.0509

Supplemental Table 9. Results from a multivariable linear regression examining the relationship between Endotrophin (ETP) plasma levels and HIV status, sex, race, age, and whole-body percent fat (%BF).

**Predictor**	**β**	**95% CI**	**P value**
HIV status (PWH vs PWoH)	0.96	(0.28, 1.64)	*0.006*
Sex (male vs female)	0.00	(−0.76, 0.76)	1.000
Age (years)	0.02	(−0.003, 0.046)	0.079
Body fat (%)	0.04	(−0.003, 0.085)	0.068
Race (Black vs White)	−0.35	(−1.23, 0.53)	0.432
Race (Other vs White)	−0.45	(−1.08, 0.17)	0.152

Multivariate OLS regression of plasma endogenous thrombin potential (ETP) on HIV status, adjusted for sex, age, percent body fat, and race. ETP was Box-Cox transformed ($\lambda = 0.29$) to satisfy normality and homoscedasticity assumptions prior to model fitting. β coefficients represent the estimated change in transformed ETP per unit increase in each continuous predictor or relative to the reference category for categorical variables. Reference categories: HIV-negative (PWoH) for HIV status, female for sex, and White for race. "Other" race includes Asian, Native Hawaiian/Pacific Islander, and more than one race. N = 119. Statistically significant associations ($p < 0.05$) are italicized.

Supplemental Table 10. Multivariable linear regression models restricted to people with HIV (PWH) examining associations between plasma endotrophin (ETP) levels, antiretroviral treatment characteristics, adiposity and demographic factors.

Predictor	Model 1		Model 2		Model 3		Model 4	
	**β (95% CI)**	**p-value**	**β (95% CI)**	**p-value**	**β (95% CI)**	**p-value**	**β (95% CI)**	**p-value**
Legacy d-drug exposure (yes vs no)	0.18 (-0.27, 0.62)	0.44	0.24 (-0.23, 0.71)	0.31	0.08 (-0.36, 0.53)	0.70	0.19 (-0.27, 0.65)	0.41
Current INSTI use (yes vs no)	-0.01 (-0.58, 0.55)	0.96	0.00 (-0.57, 0.57)	0.99	0.04 (-0.52, 0.59)	0.89	0.02 (-0.56, 0.61)	0.93
CD4+ T-cell count	0.0003 (-0.0003, 0.0009)	0.37	0.0003 (-0.0003, 0.0009)	0.27	0.0003 (-0.0003, 0.0008)	0.35	0.0003 (-0.0003, 0.0009)	0.33
%BF	0.01 (-0.02, 0.04)	0.39	0.02 (-0.01, 0.06)	0.21	0.01 (-0.02, 0.04)	0.38	0.01 (-0.02, 0.04)	0.50
Sex (Male vs Female)	—	—	0.30 (-0.29, 0.89)	0.31	—	—	—	—
Age (years)	—	—	—	—	0.02 (-0.00, 0.04)	0.06	—	—
Race: Black (vs White)	—	—	—	—	—	—	-0.10 (-0.53, 0.34)	0.66
Race: Other (vs White)	—	—	—	—	—	—	-0.41 (-1.24, 0.42)	0.32

Model 1 (base): legacy d-drug exposure, current INSTI use, CD4+ T-cell count, and %BF. Model 2: Model 1 + sex. Model 3: Model 1 + age. Model 4: Model 1 + race. ETP was Box-Cox transformed ($\lambda = 0.09$) prior to model fitting. β coefficients represent the estimated change in transformed ETP per unit increase in each continuous predictor or relative to the reference category for categorical variables. Reference categories: no legacy d-drug exposure, no INSTI use, female for sex, White for race. “Other” race includes Asian, Native Hawaiian/Pacific Islander, and more than one race. — indicates variable not included in that model. Statistically significant associations ($p < 0.05$) are italicized.

Supplemental Table 11. Adiposity-stratified multivariable linear regression models examining associations between HIV status and plasma endotrophin (ETP) levels among individuals with normal body fat (NBF) and high body fat (HBF).

**Predictor**	**NBF (n = 51)**		**HBF (n = 68)**	
	**β (95% CI)**	**p-value**	**β (95% CI)**	**p-value**
**HIV status**	0.98 (0.03, 2.00)	*0.037*	0.63 (−0.31, 1.56)	0.186
**Sex (Male)**	0.48 (−0.97, 1.93)	0.513	0.66 (−0.60, 1.91)	0.299
**Age**	0.03 (−0.01, 0.06)	0.120	0.02 (−0.01, 0.05)	0.238
**Race: Black**	−0.97 (−2.28, 0.34)	0.143	0.18 (−1.01, 1.36)	0.768
**Race: Other**	−0.45 (−1.40, 0.50)	0.344	−0.56 (−1.42, 0.29)	0.192
**%BF**	0.05 (−0.09, 0.18)	0.463	*0.13 (0.04, 0.21)*	*0.005*

Multivariate OLS regression of Box-Cox–transformed plasma (ETP) on HIV status, stratified by body fat group and adjusted for sex, age, %BF, and race. ETP was Box-Cox transformed ($\lambda = 0.27$ for NBF, $\lambda = 0.30$ for HBF) to satisfy normality and homoscedasticity assumptions prior to model fitting. β coefficients represent the estimated change in transformed ETP per unit increase in each continuous predictor or relative to the reference category for categorical variables. Reference categories: HIV-negative (PWoH) for HIV status, female for sex, and White for race. "Other" race includes Asian, Native Hawaiian/Pacific Islander, and more than one race. Statistically significant associations ($p < 0.05$) are italicized.

Supplemental References

1. Bredella MA, et al. Assessment of abdominal fat compartments using DXA in premenopausal women from anorexia nervosa to morbid obesity. *Obesity* . 2013;21(12):2458–2464.

2. Kaul S, et al. Dual-Energy X-Ray Absorptiometry for Quantification of Visceral Fat. *Obesity*. 2012;20(6):1313–1318
